# Advanced Electrode Materials for Water Electrolysis: Design Principles, Performance Trade-Offs, and Technology Pathways Across ALK, PEM, SOEC, and AEM Systems

**DOI:** 10.3390/ma19112259

**Published:** 2026-05-26

**Authors:** Bożena Łosiewicz

**Affiliations:** Institute of Materials Engineering, Faculty of Science and Technology, University of Silesia in Katowice, 41-500 Chorzów, Poland; bozena.losiewicz@us.edu.pl; Tel.: +48-32-3497-527

**Keywords:** green hydrogen, water electrolysis, electrode materials, electrocatalysts, hydrogen evolution reaction, oxygen evolution reaction, ALK electrolysis, PEM electrolysis, SOEC, AEM electrolysis

## Abstract

**Highlights:**

**What are the main findings?**

- Rapid growth of research on electrode materials for water electrolysis technologies between 2021 and 2025.
- Transition-metal-based catalysts and heterostructured materials show promising activity comparable to noble-metal catalysts.
- Each electrolysis technology (ALK, PEM, SOEC, AEM) requires specific electrode materials due to different operating environments and reaction mechanisms.

**What are the implications of the main findings?**

- Advanced catalyst design and interface engineering can significantly improve electrolysis efficiency and durability.
- Reducing noble metal loading is essential for lowering electrolyzer costs and enabling large-scale hydrogen production.
- Integrated materials research and system-level optimization will be critical for accelerating the commercialization of green hydrogen technologies.

**Abstract:**

The transition toward low-carbon energy systems has intensified interest in sustainable hydrogen production technologies. One of the most promising methods for producing green hydrogen is water electrolysis powered by renewable energy. This work reviews recent advances in electrode materials used in four major electrolysis technologies: alkaline (ALK), proton exchange membrane (PEM), solid oxide electrolysis cells (SOEC), and anion exchange membrane (AEM). A bibliometric analysis of scientific publications from 2021 to 2025 highlights the rapid growth of research and the increasing importance of electrode materials in improving electrolysis performance. Operating environments, material requirements, and catalytic properties are compared across these systems. Recent developments in electrocatalysts—including transition-metal alloys, heterostructured catalysts, defect-engineered materials, and nanostructured systems—are evaluated in terms of catalytic activity, durability, and scalability. Particular attention is given to reducing noble metal usage while maintaining high electrochemical performance. Results indicate that transition-metal-based catalysts and engineered interfaces can achieve activity comparable to noble-metal systems while offering better cost efficiency. However, challenges related to long-term durability, large-scale synthesis, and standardized testing persist. Continued interdisciplinary research in materials design and electrochemical engineering is essential to enable efficient, durable, and cost-effective green hydrogen production.

## 1. Introduction

The global shift toward carbon-neutral energy systems has accelerated research into sustainable hydrogen production, with green hydrogen emerging as a key component of low-carbon energy systems [1,2,3,4,5]. Produced via water electrolysis powered by renewable electricity, it provides a zero-carbon pathway and enables decarbonization of energy-intensive sectors such as heavy industry, long-distance transport, and high-temperature chemical processes—unlike fossil-based blue hydrogen with carbon capture [4,5,6,7,8]. It also functions as a flexible energy carrier, supporting energy storage, sector coupling, and grid stabilization [9,10,11]. It can be used in fuel cells, ammonia and methanol synthesis, and low-carbon steel production [6,10,12]. However, large-scale deployment remains limited by electricity costs, capital expenditure, durability, and efficiency, despite growing competitiveness in regions rich in renewables [11,12].

Among production methods, water electrolysis remains the most mature and scalable technology [13,14,15,16,17]. Compared with emerging alternatives such as photoelectrochemical, thermochemical, or biological routes, electrolysis provides a direct and controllable link between renewable power and hydrogen generation [16,17,18,19,20,21]. Ongoing advances in materials and system engineering continue to enhance its efficiency, flexibility, and durability, reinforcing its central role in the hydrogen economy [22,23,24,25,26]. Recent review articles have also highlighted rapid progress in advanced electrode materials, membrane engineering, and interface optimization for water electrolysis technologies [27,28].

Four major electrolysis platforms currently dominate research and development efforts: alkaline electrolysis (ALK), proton exchange membrane (PEM) electrolysis, solid oxide electrolysis (SOEC), and anion exchange membrane (AEM) electrolysis [13,25]. Each system operates under distinct electrochemical environments, temperature regimes, and material constraints, which directly influence electrode design, catalyst selection, and long-term stability [29,30,31,32]. Consequently, electrode materials have become one of the most critical research areas, as they determine catalytic activity, overpotential, degradation behavior, and overall system performance [25].

**Figure 1:**
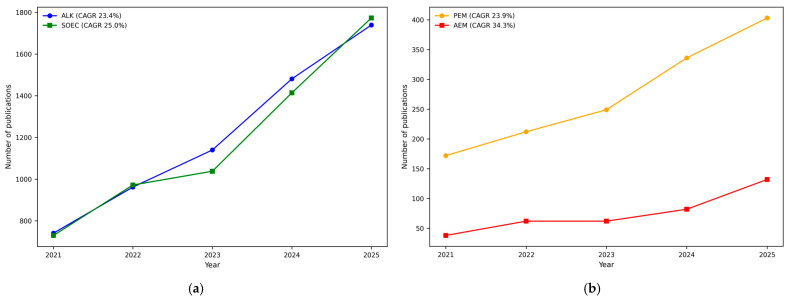
Growth of publications on water electrolysis technologies from 2021 to 2025 based on the Scopus database search conducted on 21 February 2026: (**a**) High-maturity systems (ALK—alkaline, SOEC—solid oxide electrolysis cell); (**b**) Emerging membrane-based systems (PEM—proton exchange membrane, AEM—anion exchange membrane).

To quantitatively assess research dynamics in this field, a bibliometric analysis was performed using the Scopus database on 21 February 2026 (Figure 1). The search employed TITLE-ABS-KEY queries combining water electrolysis technologies with the terms “electrode,” “electrode material,” and “catalyst,” and was limited to peer-reviewed articles and review papers. Between 2021 and 2025, a substantial increase in publications related to electrode materials for water electrolysis was observed. For ALK systems, the number of publications rose from 741 to 1739; for SOEC, from 730 to 1773; and for PEM, from 172 to 403. Although AEM technologies showed lower absolute values (38 to 132), they exhibited the highest relative growth dynamics. The compound annual growth rate (CAGR) reached approximately 23.8% for ALK, 24.8% for SOEC, 23.7% for PEM, and 36.5% for AEM. In structural terms, ALK and SOEC together accounted for approximately 87% of all publications over the analyzed period (44.3% and 43.4%, respectively), while PEM represented around 10% and AEM 2.3%. Despite its smaller share, AEM demonstrated the most rapid expansion, indicating increasing scientific interest in this technology.

Year-to-date data for 2026 (as of 21 February) further confirm the continuation of this upward trend. A total of 415 publications were indexed for ALK, 421 for SOEC, 83 for PEM, and 30 for AEM, corresponding to approximately 21–24% of the respective 2025 annual totals. These lower values are attributable to incomplete annual indexing rather than a slowdown in research activity. When normalized to the fraction of the year covered, the data indicate sustained growth across all electrolysis platforms.

The mature–emerging distinction highlights shifting research priorities. Established technologies (ALK, SOEC) focus mainly on incremental improvements in durability, catalyst optimization, electrode design, and system integration, while PEM advances through membrane–electrode assembly refinement and reduced noble-metal usage [13,25,29]. In contrast, emerging AEM systems emphasize novel materials, interface engineering, and minimizing precious metals while maintaining high activity [22,30]. Across all platforms, rapid publication growth underscores the pivotal role of electrode materials in lowering hydrogen production costs and enhancing efficiency [12,33].

Despite extensive progress, key gaps remain. Long-term durability under industrial conditions is insufficiently understood, and many laboratory-scale studies lack transferability to commercial systems [29,34]. Scalable, sustainable synthesis routes and standardized testing protocols are still needed [22,31]. Advanced in situ and operando characterization, along with stronger integration of materials science, electrochemistry, and engineering, are essential to address multiscale challenges [25]. Computational modeling and data-driven approaches are increasingly important for accelerating material discovery [35,36,37].

Additional challenges arise from coupling electrolysis with intermittent renewables, which impose dynamic stresses on electrodes [35,36,37,38]. Future strategies must improve intrinsic activity and stability under variable conditions through electronic structure tuning, defect engineering, heterostructures, 3D architectures, and improved membrane–electrode integration [25,32,39].

Given the rapid expansion of research and the strategic role of water electrolysis in the green hydrogen economy [30,33,40,41], a comprehensive cross-platform review is timely. This work systematically compares recent advances in ALK, PEM, SOEC, and AEM systems (2021–2025), evaluating electrode material requirements, catalyst design strategies, performance metrics, and technological challenges across these platforms.

Although research on water electrolysis has grown rapidly, many studies focus on individual technologies, particularly ALK or PEM electrolysis. Direct comparisons of electrode materials, catalytic performance, and operational constraints across all major electrolysis systems remain relatively limited. Moreover, a significant portion of the literature emphasizes catalytic activity under laboratory conditions, while less attention is given to durability, scalability, and system-level factors that are critical for industrial hydrogen production.

This review integrates bibliometric analysis with a systematic evaluation of catalyst design strategies, electrochemical performance, and material stability. By linking advances in materials science with device- and system-level requirements, it highlights emerging trends in catalyst development and identifies key technological gaps. The study also aims to clarify structure–property–performance relationships and promote more standardized evaluation methods [15,25,26], providing a strategic roadmap for scalable, durable, and cost-effective green hydrogen production. Several recent review articles have also highlighted the rapid development of electrode materials and electrolysis technologies, emphasizing the importance of catalyst design, system integration, and cost reduction strategies [25,26,27,28,29,30,31,32,33,39]. These studies complement the present work by focusing on specific technologies or material classes, whereas this review provides a cross-platform comparison of ALK, PEM, SOEC, and AEM systems.

Generative artificial intelligence (GenAI) tools were used solely to assist in the preparation of schematic graphical illustrations and the graphical abstract included in this review. OpenAI ChatGPT with the DALL·E image generation model was employed to support visualization and graphical design. All scientific interpretation, validation, and final editing were performed exclusively by the author. The author reviewed and verified all AI-assisted content and takes full responsibility for the accuracy and originality of the final material.

## 2. Background of Analysis

### 2.1. Fundamentals of Water Electrolysis Technology

#### 2.1.1. ALK Electrolysis

**Figure 2:**
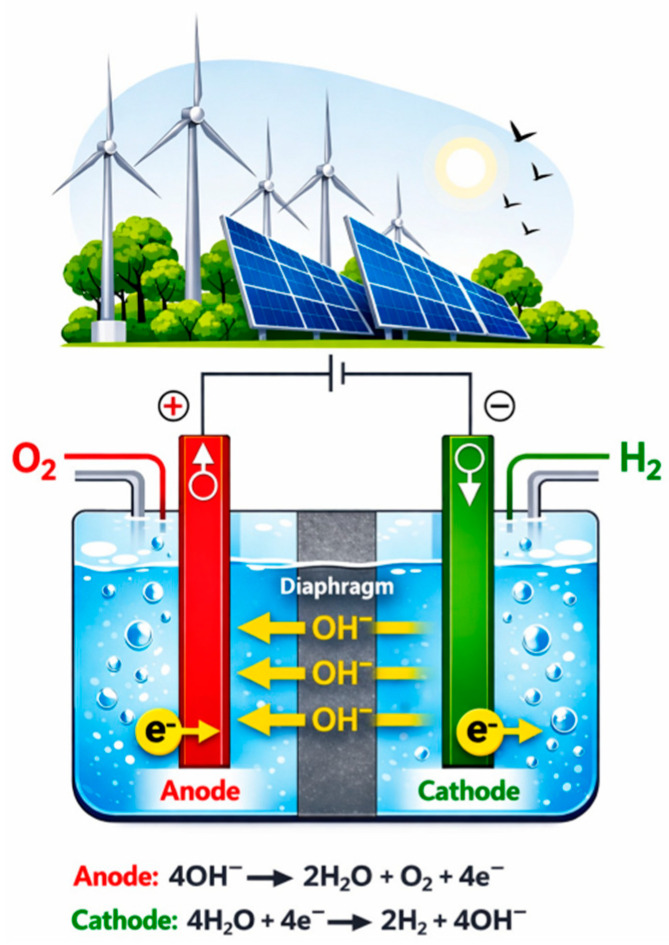
Schematic overview of alkaline (ALK) water electrolysis technology integrated with renewable energy sources (RES).

ALK is one of the most mature and industrially established routes for green hydrogen production, particularly when coupled with renewable energy sources (RES) such as wind and solar power [2,3,9,13,15,22,30,38]. Due to decades of industrial deployment, ALK systems have reached a high technology readiness level (TRL 9) and are widely regarded as reliable and scalable [4,13,14,33,42,43,44,45,46,47]. A schematic representation of an ALK system integrated with RES is presented in Figure 2.

In ALK electrolysis, water splitting occurs in a liquid electrolyte, typically 25–40 wt% KOH or NaOH, which ensures high ionic conductivity and efficient transport of hydroxide ions (OH^−^) between electrodes [13,43,45,46]. The alkaline environment enables long-term electrode stability and the use of non-noble metal catalysts [25,42,43,45]. In contrast to PEM systems, charge transport in ALK proceeds via OH^−^ migration through the electrolyte and diaphragm, while electrons are transferred through the external circuit [13,43,45,46]. The half-reactions under alkaline conditions correspond to the hydrogen evolution reaction (HER) at the cathode and the oxygen evolution reaction (OER) at the anode. The mechanistic aspects of HER and OER in alkaline media, including Volmer–Heyrovsky/Tafel pathways and multistep oxygen intermediate formation, have been extensively discussed in the literature [42,43,44,45,47].

The electrodes are physically separated by a porous diaphragm that enables ionic conduction while preventing gas crossover and recombination, thereby ensuring operational safety and hydrogen purity [13,25,43,45,46]. Separator properties such as ionic conductivity, mechanical robustness, and chemical resistance critically affect internal resistance and overall system efficiency. Modern separator materials are therefore engineered to minimize ohmic losses and gas permeability while maintaining long-term durability under highly alkaline conditions [43,45,46].

A key advantage of ALK technology lies in the use of earth-abundant, non-noble metal electrodes, which significantly reduces capital costs compared to noble-metal-based systems [2,25,42,45,46]. Nickel and nickel-based alloys remain the dominant electrode materials due to their excellent corrosion resistance in concentrated alkaline media, good electrical conductivity, and favorable catalytic properties [25,42,43,45]. However, the intrinsically slower kinetics of HER and particularly OER in alkaline environments necessitate catalyst optimization and surface engineering strategies [42,43,44,45,47].

Synthesis strategies play a decisive role in determining catalytic performance. Techniques such as nanostructuring, defect engineering, heterostructure fabrication, and controlled doping enable precise tuning of the electronic structure and surface energetics, leading to improved HER/OER activity and charge-transfer characteristics [42,43,44,45]. Common synthesis routes include solvothermal/hydrothermal methods, electrodeposition, chemical vapor deposition, and atomic layer deposition, which allow control over morphology, crystallinity, and active site density. However, durability is strongly dependent on structural stability under operating conditions. Therefore, future synthesis approaches should focus on scalable, reproducible methods that preserve catalyst integrity and mitigate degradation phenomena such as dissolution, agglomeration, and phase transformation [33,39].

Recent advances focus on alloying, nanostructuring, heterointerface design, and electronic structure modulation to optimize adsorption energies of reaction intermediates [42,44,47,48]. For example, NiMo alloys exhibit enhanced HER activity by tuning hydrogen binding energy and facilitating water dissociation kinetics [44,45]. Similarly, NiFe-based oxides and layered double hydroxides (LDH) demonstrate superior OER performance due to synergistic electronic interactions and optimized oxygen intermediate adsorption/desorption energetics [42,47,48]. Operando studies and theoretical calculations further support the role of dynamic surface reconstruction and active phase evolution during alkaline OER [42,45,47]. The comprehensive material requirements for ALK electrodes are summarized in Table 1.

ALK electrolyzers typically operate at temperatures from ambient up to 80–90 °C and at pressures up to several MPa [13,29,43,45,46]. Elevated temperature improves reaction kinetics, reduces activation overpotentials, and enhances ionic conductivity of the electrolyte, leading to lower cell voltages and improved system efficiency [43,45,46]. Industrial current densities commonly exceed 200 mA cm^−2^, with ongoing research targeting even higher values while maintaining long-term electrochemical durability [43,45,46]. Advanced modeling approaches and electrochemical impedance analyses contribute to understanding electrolyte conductivity and dynamic response under variable loads [45,46].

Despite its technological maturity, ALK electrolysis exhibits certain limitations related to dynamic operation and system integration [13,30,39,45]. Compared to PEM systems, ALK units generally display slower start-up times and lower load-following flexibility, which may constrain direct coupling with highly intermittent RES without additional power management strategies [30,35,36,37,38,39]. Furthermore, the presence of a liquid electrolyte and diaphragm contributes to increased internal resistance and potential gas crossover, affecting efficiency and long-term durability [43,45,46].

Nevertheless, ALK remains highly competitive for large-scale hydrogen production due to its relatively low capital expenditure, established industrial supply chains, and reliance on abundant transition metals [2,30,43,45]. When powered by renewable electricity, ALK electrolysis enables low-carbon hydrogen generation and supports the decarbonization of energy-intensive sectors such as ammonia synthesis, refining, metallurgy, and heavy-duty transport [2,6,10,30,40]. Continuous advances in catalyst development, separator materials, system modeling, and sector coupling strategies are expected to further enhance efficiency, durability, and economic viability, reinforcing the central role of ALK technology in the emerging hydrogen economy [33,37,43,45,46].

#### 2.1.2. PEM Electrolysis

**Figure 3:**
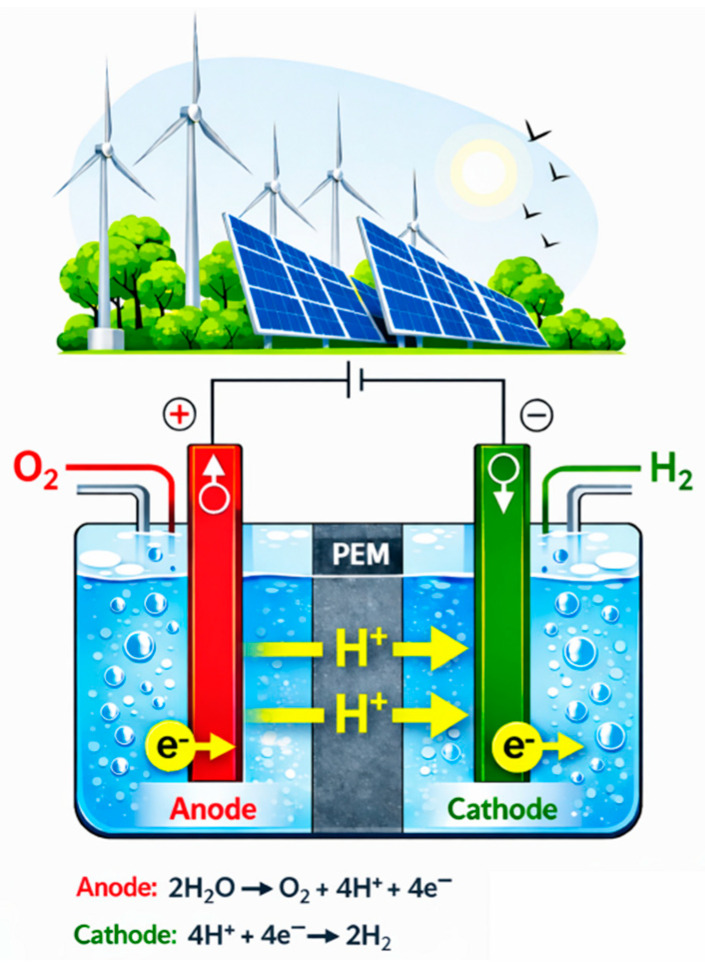
Schematic overview of proton exchange membrane (PEM) water electrolysis technology integrated with renewable energy sources (RES).

PEM electrolysis is one of the most advanced technologies for green hydrogen production within the broader hydrogen economy framework [1,2,3,4,5,9,10,30,39,40]. In PEM electrolyzers, the electrolyte is a solid proton-conducting polymer membrane, typically based on perfluorosulfonic acid (PFSA) materials such as Nafion, which ensures selective proton transport from the anode to the cathode while preventing electron conduction and gas crossover [49,50,51,52,53]. As illustrated in Figure 3, when direct current (DC) is applied, water is oxidized at the anode to generate oxygen, protons, and electrons. The electrons travel through the external circuit, whereas the protons migrate across the membrane and are reduced at the cathode to form high-purity hydrogen gas. This solid-state architecture eliminates the need for liquid alkaline electrolytes and enables compact cell design with high current density operation [13,14,15,16,17,45,53].

PEM electrolysis operates typically below 80 °C and can reach operating pressures of 30–40 bar, offering advantages in downstream hydrogen compression and system integration [45,51,53]. Compared to ALK systems [42,43,44,45,46,47,48], PEM electrolyzers show lower ohmic resistance, superior dynamic response, and improved partial-load performance, which are particularly valuable in coupling with intermittent RES such as solar and wind power, as shown in Figure 3. The rapid response time and operational flexibility make PEM electrolyzers highly suitable for power-to-hydrogen applications in integrated multi-energy systems, microgrids, and hydrogen–power–transportation networks [35,36,37]. Consequently, PEM technology is increasingly viewed as a core enabler of decarbonization strategies aligned with Net Zero objectives [1,4,5,10,40].

It operates in a strongly acidic environment, which imposes strict material requirements on membranes, catalysts, and bipolar plates, as summarized in Table 2. High proton conductivity at the catalyst–membrane interface is essential to minimize interfacial resistance, and PFSA-based catalyst layers remain the benchmark solution due to their chemical compatibility and ionic transport properties [49,50,52]. The OER at the anode requires highly corrosion-resistant catalysts, typically iridium oxide (IrO_2_) or ruthenium oxide (RuO_2_), while platinum-based materials dominate the HER at the cathode [25,52,53,54,55,56]. These noble metals provide ultra-low overpotentials and high exchange current densities, ensuring high energy efficiency [25,45], but they also significantly increase system cost and raise concerns regarding critical raw material availability [11,12,33,41]. Efforts to reduce noble metal loading through nanostructuring, sputtering techniques, and core–shell architectures are actively being pursued [52,53,54,55,56].

Although PEM electrolysis offers high hydrogen purity, compactness, and operational flexibility, several durability challenges remain. Membrane degradation and hydrogen crossover through catalyst-coated PFSA membranes can lead to performance losses and localized hot spots, ultimately shortening stack lifetime [49]. Catalyst dissolution under high anodic potentials and mechanical stresses associated with differential pressure and thermal cycling further affects long-term stability [45,52,53]. Efficient thermal management is required to maintain stable operation within the optimal temperature range and to prevent accelerated degradation. Advanced modeling and control strategies, including power electronics integration and machine-learning-based optimization, are increasingly being investigated to enhance efficiency and operational stability [57,58].

From a system perspective, PEM electrolysis complements other hydrogen production pathways, including ALK electrolysis [42,43,44,45,46,47,48], high-temperature electrolysis [32], and biohydrogen routes [18,19,20,21,22,23,24], contributing to diversified hydrogen supply chains [28,29]. Its compatibility with RES and its fast dynamic response position it as a preferred technology in emerging hydrogen infrastructure development and large-scale deployment scenarios [33,34,35,51]. Market analyses indicate strong projected growth in electrolyzer manufacturing capacity driven by global hydrogen demand [33,41], while industrial-scale testing and standardization remain critical to accelerating commercialization [34].

Overall, as depicted in Figure 3 and detailed in the material performance criteria of Table 2, PEM water electrolysis represents a technologically mature yet rapidly evolving platform that combines high efficiency, high-purity hydrogen output, and excellent renewable integration capability. Addressing challenges related to catalyst cost, membrane durability, and large-scale manufacturing will be decisive for achieving economically competitive green hydrogen production and supporting global sustainability and climate targets [1,2,3,4,5,11,12,39,40,41,52,53].

#### 2.1.3. SOEC

**Figure 4:**
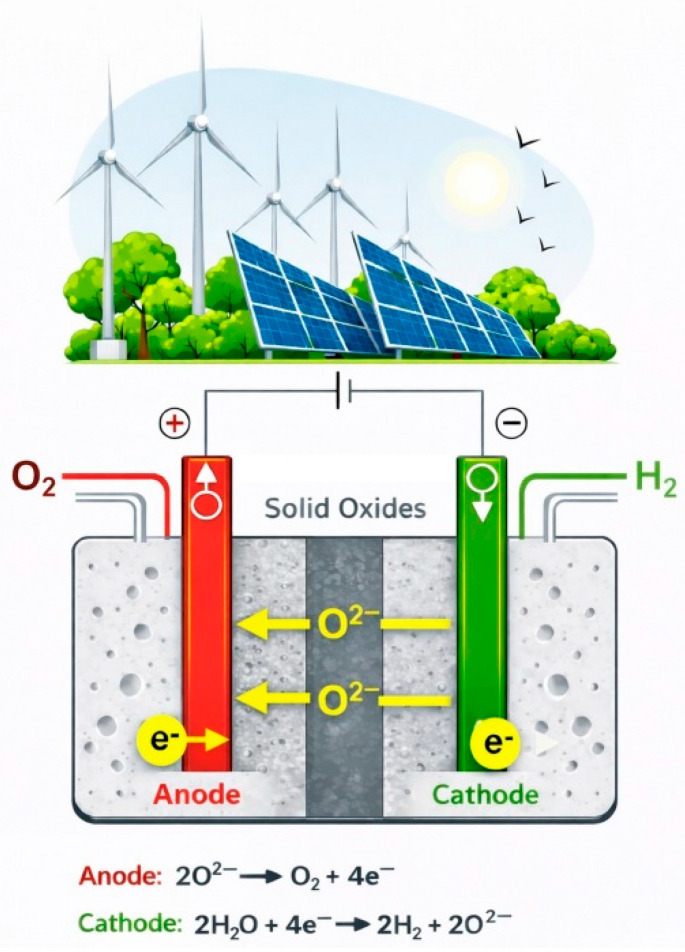
Schematic overview of solid oxide electrolysis cell (SOEC) technology integrated with renewable energy sources (RES).

SOEC cells operate at elevated temperatures and employ a dense ceramic electrolyte capable of conducting oxygen ions (O^2−^), as illustrated in Figure 4. In electrolysis mode, oxygen ions migrate from the fuel electrode (cathode) to the oxygen electrode (anode), where they recombine to form molecular oxygen and release electrons to the external circuit. The electrolyte is typically based on yttria-stabilized zirconia (YSZ), which exhibits high oxygen-ion conductivity at elevated temperatures due to the presence of oxygen vacancies in its fluorite crystal structure [59,60,61,62,63,64,65]. Alternative materials, such as doped ceria systems, have also been investigated to improve ionic conductivity and mitigate degradation processes [60,65].

SOEC is considered an advanced high-temperature electrolysis technology for hydrogen production from steam and for co-electrolysis of steam and carbon dioxide to produce syngas (H_2_ + CO), which can subsequently be used for synthetic fuel synthesis [32,66,67]. In contrast to low-temperature technologies such as PEM and ALK electrolysis, SOEC typically operates in the range of 500–1000 °C, with practical systems most often functioning between 700 and 850 °C in order to balance efficiency and long-term durability [66,68,69]. The elevated operating temperature reduces the electrical energy demand by supplying part of the reaction enthalpy in the form of heat, thereby improving thermodynamic efficiency and lowering activation overpotentials [32,66].

An SOEC consists of a dense solid electrolyte sandwiched between two porous electrodes that provide extensive triple-phase boundary (TPB) regions for electrochemical reactions. The electrolyte is a gas-tight, non-porous metal oxide, most commonly ZrO_2_ doped with Y_2_O_3_, which ensures selective oxygen-ion transport while separating the product gases [59,60,65]. When a DC voltage is applied, steam (or a mixture of steam and CO_2_ in co-electrolysis mode) is introduced to the fuel electrode. At this electrode, steam is electrochemically reduced to hydrogen and oxygen ions, while CO_2_ can be reduced to CO under co-electrolysis conditions. The generated O^2−^ ions migrate through the electrolyte toward the oxygen electrode, where they are oxidized to form O_2_ gas. A schematic overview of SOEC water electrolysis integrated with RES is presented in Figure 4, highlighting the potential coupling of electrical and thermal inputs in hybrid energy systems [66,70].

High-temperature operation offers several strategic advantages, particularly in industrial contexts where excess waste heat is available. The possibility of utilizing industrial waste heat or concentrated solar thermal energy improves overall system efficiency and reduces the specific electricity consumption per kilogram of hydrogen produced [66,70,71]. Furthermore, SOEC enables operational flexibility by accommodating different feedstocks, including steam, carbon dioxide, or their mixtures, thereby facilitating integration with carbon capture and utilization (CCU) pathways and Power-to-X (PtX) platforms [32,67,71]. PtX refers to a group of technologies that convert electrical energy—typically from renewable sources—into other forms of energy carriers or chemicals, such as hydrogen (Power-to-H_2_), synthetic fuels (Power-to-Fuels), ammonia (Power-to-NH_3_), or heat (Power-to-Heat), enabling energy storage, sector coupling, and decarbonization of hard-to-abate sectors. The direct production of syngas with adjustable H_2_/CO ratios is particularly attractive for Fischer–Tropsch synthesis and synthetic fuel production. The H_2_/CO ratio is primarily controlled by operating conditions, such as temperature, applied voltage, and the steam-to-CO_2_ feed ratio, while electrode materials mainly influence catalytic activity and selectivity [66,70,71].

Despite these advantages, SOEC technology faces significant materials and durability challenges associated with prolonged operation at high temperatures. Degradation mechanisms include nickel migration in Ni–YSZ fuel electrodes, microstructural coarsening, delamination at electrode–electrolyte interfaces, redox instability during transient operation, and thermomechanical stresses resulting from thermal cycling [61,62,63,64,65,72,73]. Ni migration and related microstructural evolution phenomena have been extensively analyzed, leading to revised degradation hypotheses and advanced modeling approaches [63,65]. The composition of sweep gases and operating parameters further influence degradation rates, particularly at the oxygen electrode [61,73]. Consequently, the development of durable, redox-stable, and thermally compatible materials remains a critical research priority.

The key functional and materials requirements for SOEC electrodes and cell components are summarized in Table 3, which outlines the critical physicochemical properties necessary to ensure efficient and stable operation under high-temperature electrolysis conditions.

SOEC is considered highly promising for large-scale hydrogen and syngas production, especially in industrial sectors requiring high-temperature process heat. Potential applications include hydrogen supply for fuel cells, feedstock provision for ammonia and methanol synthesis, and integration into multi-energy systems combining electricity, heat, and gas infrastructures [32,66,71]. However, compared with PEM and ALK electrolysis, SOEC technology remains at a lower maturity level and requires further advances in stack design, degradation mitigation, and cost reduction to achieve widespread commercialization [69,72]. Ongoing research focuses on optimizing electrolyte and electrode materials, improving thermal expansion compatibility, enhancing catalytic activity for H_2_O/CO_2_ reduction, and ensuring long-term structural integrity under high-temperature operation, as summarized in Table 3 [59,60,61,62,63,64,65,68,69].

#### 2.1.4. AEM Electrolysis

**Figure 5:**
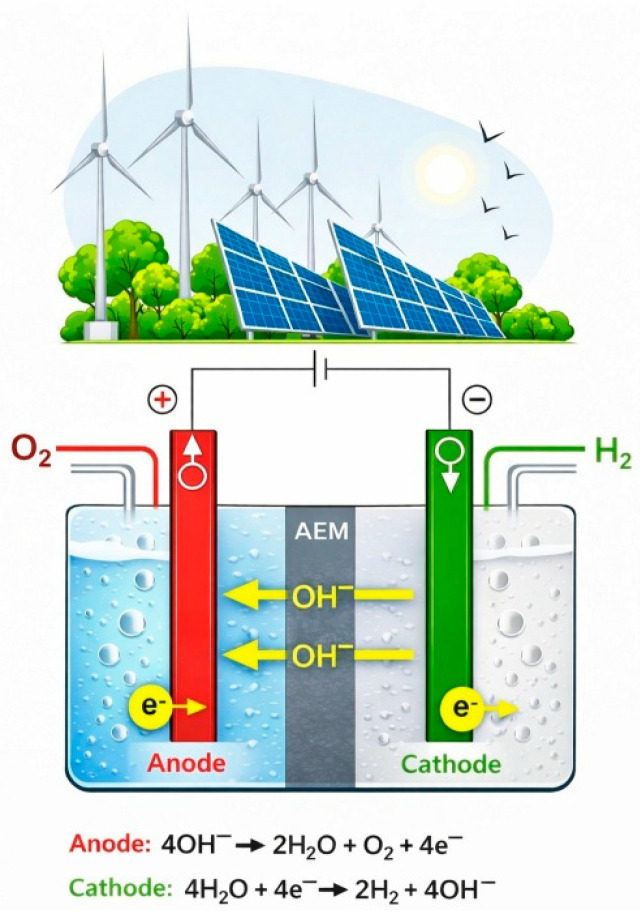
Schematic overview of anion exchange membrane (AEM) water electrolysis technology integrated with renewable energy sources (RES).

AEM electrolyzers employ an anion-conducting polymer membrane that transports hydroxide ions (OH^−^) from the cathode to the anode. At the cathode, water is reduced to hydrogen and hydroxide ions, while at the anode, hydroxide ions are oxidized to oxygen and water (Figure 5). The AEM is typically composed of a polymer backbone functionalized with positively charged groups (e.g., quaternary ammonium), enabling selective transport of OH^−^ ions while blocking electrons and product gases [74,75,76,77].

AEM electrolysis is an emerging technology combining features of ALK and PEM systems [13,74,75]. Similar to PEM electrolysis, it uses a solid polymer membrane to separate the anode and cathode compartments; however, it operates in an alkaline environment and conducts hydroxide ions instead of protons. When DC is applied, the cathodic HER produces H_2_ and OH^−^ ions. The hydroxide ions migrate through the membrane to the anode, where the OER generates O_2_ and water [74,75,76,77].

The membrane is a key functional component, providing ionic conductivity and separation of reactant and product gases. Electrode materials in AEM electrolyzers are typically based on non-precious transition metals, such as Ni, NiFeOx, or Co-based catalysts, particularly for the cathode and anode in alkaline conditions [42,43,44,48]. Although noble metals (e.g., Ir-based oxides) may still be applied in some configurations, significant research efforts focus on eliminating platinum group metals (PGMs) to reduce system cost [74,78,79].

AEM systems generally operate at temperatures between 50 and 80 °C and at near-ambient or moderately elevated pressures [74,77,80]. Compared with solid oxide electrolysis (SOE), they operate at significantly lower temperatures, while their operating range is comparable to or slightly higher than that of PEM systems. A key advantage of AEM electrolysis is the potential reduction in capital cost through the use of less expensive membrane materials and PGM-free catalysts [74,77,81]. Operation in alkaline conditions enables the use of earth-abundant electrocatalysts, while moderate temperature operation can improve reaction kinetics compared to conventional alkaline systems [43,74]. Furthermore, AEM technology offers flexibility in electrolyte composition (pure water or diluted KOH), which can simplify balance-of-plant requirements [77,82].

However, several challenges remain. The long-term chemical and mechanical stability of AEMs in highly alkaline environments is a critical issue, as membranes are susceptible to nucleophilic attack and degradation [75,81,83,84]. Catalyst durability under dynamic operating conditions and the mitigation of gas crossover are additional concerns affecting lifetime and hydrogen purity [75,83]. Consequently, AEM electrolysis is less technologically mature than PEM and conventional ALK electrolysis and requires further research to improve durability and scale-up readiness [74,75,76].

AEM electrolysis is currently being investigated for renewable energy integration, decentralized hydrogen production, and industrial-scale green hydrogen generation [33,38,39]. Owing to its cost-reduction potential and improving performance metrics, it is regarded as a promising pathway toward competitive green hydrogen production.

In practical and industrial operating conditions, the long-term durability of AEM electrolyzers remains a critical bottleneck compared to established ALK systems. Unlike ALK technology, where electrode and separator materials have demonstrated stable operation over extended lifetimes [13,25,43,45], AEM membranes are susceptible to chemical degradation mechanisms such as nucleophilic attack by OH^−^ ions, backbone cleavage, and loss of functional groups [75,81,83,84]. These processes lead to decreased ionic conductivity, increased gas crossover, and overall performance degradation.

Furthermore, under dynamic operating conditions typical of renewable energy coupling, AEM systems may experience additional mechanical and chemical stress, accelerating membrane failure [35,36,37,38,75,83]. In contrast, ALK systems benefit from robust liquid electrolytes and well-established material stability, making them more reliable for large-scale industrial deployment [13,25,43,45]. Therefore, improving membrane durability and chemical stability remains one of the key challenges limiting the commercialization of AEM electrolysis [74,75,76,83,84].

Each electrolysis technology operates under distinct physicochemical conditions and therefore requires electrode and membrane materials with tailored properties. The key material requirements specific to AEM electrolysis are summarized in Table 4.

ALK electrolyzers are a mature and commercially established technology, but they require concentrated KOH solutions and may suffer from corrosion-related issues [46]. PEM electrolyzers achieve high current densities and compact system design; however, they rely on expensive noble-metal catalysts and face membrane degradation challenges [51,52,53]. SOEC offers high electrical efficiency due to elevated operating temperatures, but demands advanced materials resistant to thermal and redox stresses [66,69].

In comparison, AEM electrolyzers aim to combine the material cost advantages of ALK systems with the compact architecture of PEM technology. While ALK and PEM systems are commercially available, SOEC and AEM technologies are still progressing from pilot and laboratory scales toward industrial deployment and are expected to play a significant role in the future hydrogen economy [33,74,75,76].

### 2.2. Global Electrolyzer Manufacturing Capacity

**Figure 6:**
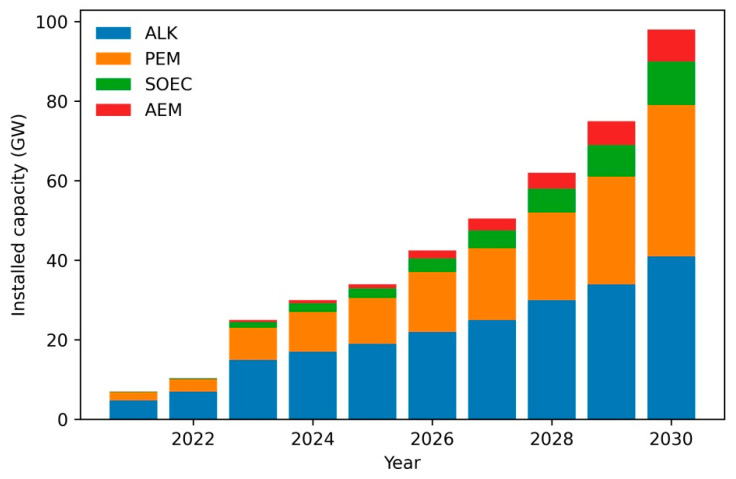
Global installed electrolyser capacity by technology (ALK—alkaline, PEM—proton exchange membrane, SOEC—solid oxide electrolysis cell, AEM—anion exchange membrane) between 2021 and 2030 developed based on [86,87,88,89,90,91,92,93,94,95].

The results presented in Figure 6 demonstrate a rapid and sustained expansion of global installed electrolyser capacity between 2021 and 2030 [86,87,88,89,90,91,92,93,94,95]. Total capacity increases from 8 GW in 2021 to 98 GW in 2030, corresponding to more than a twelvefold growth within less than a decade. This dynamic development reflects the transition of water electrolysis from early commercial deployment to large-scale industrialization, driven by climate policy targets, growing demand for low-carbon hydrogen, and declining technology costs [33,86,87,88,89,90,93]. The acceleration of capacity additions observed after 2024 (Figure 6) indicates the entry of multi-hundred-megawatt and gigawatt-scale projects, consistent with international hydrogen deployment scenarios reported in the recent literature [86,87,88,89,90,93].

ALK electrolysis remains the dominant technology in absolute terms throughout the analyzed period, with installed capacity increasing from 4.8 GW to 41 GW by 2030 (Figure 6). Despite this substantial growth, the relative share of ALK systems declines from 60% to 42%, indicating a gradual reduction in technological dominance rather than an absolute contraction. This trend reflects the high level of technological maturity, proven operational reliability, and comparatively low capital expenditure of ALK systems [13,14,42,43,44,45,46,47]. At the same time, their limited dynamic flexibility and lower suitability for direct coupling with highly variable renewable electricity sources constrain their relative competitiveness in future power systems with increasing shares of wind and solar generation [15,30,38,93].

In contrast, PEM electrolysis exhibits strong growth both in absolute and relative terms. Installed PEM capacity increases from 2 GW in 2021 to 38 GW in 2030, while its market share rises from 25% to 37%, approaching parity with ALK electrolysis by the end of the decade (Figure 6). This development highlights the increasing importance of operational flexibility, fast start-up, and load-following capability in electrolyser deployment [51,52,53,57,58]. PEM systems are particularly well suited for integration with variable RES, enabling higher utilization of surplus electricity and reduced curtailment [30,38,93]. The observed trend is therefore indicative of a system-level optimization, in which higher capital costs are increasingly offset by operational and integration benefits [12,33,39].

SOEC cells show the highest relative growth rate among all analyzed technologies. Installed SOEC capacity increases from 0.15 GW in 2021 to 11 GW in 2030, with its share rising from 2% to 11% (Figure 6). This pronounced increase suggests a transition of SOEC technology from laboratory-scale research and pilot projects toward early commercial deployment [66,67,68,69,70,72]. The growing relevance of SOEC is primarily attributed to its high electrical efficiency, particularly when integrated with high-temperature heat sources or industrial waste heat [32,66,69]. As a result, SOEC systems are increasingly considered for applications such as synthetic fuels, e-methanol, and green ammonia production, where efficiency advantages can outweigh higher system complexity and material challenges [6,66,71].

AEM electrolysis also demonstrates steady growth, with installed capacity rising from 0.05 GW to 8 GW over the analyzed period and its market share increasing from 1% to 8% (Figure 6). However, the stabilization of its relative contribution after the mid-2020s suggests cautious market adoption. AEM technology combines certain advantages of ALK and PEM systems, including reduced reliance on noble metals and improved dynamic behavior [74,75,76,77,81,84]. Nevertheless, uncertainties related to membrane durability, long-term stability, and large-scale manufacturability currently limit more rapid deployment and explain its role as a complementary rather than dominant technology [75,77,83,84,85].

Overall, Figure 6 illustrates a clear structural transformation of the electrolyser market. In 2021, deployment is heavily concentrated in a single technology, whereas by 2030, the market evolves into a diversified technological portfolio. ALK and PEM electrolysis together account for approximately 79% of installed capacity, while emerging technologies such as SOEC and AEM contribute more than 20%. This diversification reduces technology-specific risks and enables more application-oriented technology selection. Rather than converging toward a single dominant solution, the electrolyser market increasingly adopts a portfolio approach that balances cost, efficiency, flexibility, and system integration requirements [33,39,86,87,88,89,90].

From an industrial perspective, these results imply that future hydrogen projects will increasingly require tailored technology choices depending on electricity supply characteristics, heat availability, and downstream hydrogen utilization pathways [6,28,30,94]. From a policy perspective, the findings suggest that capacity-based deployment targets alone may be insufficient to ensure optimal system development. Complementary policy instruments addressing technology-specific barriers, particularly for SOEC and AEM, may be required to accelerate innovation while sustaining large-scale deployment of mature ALK and PEM systems [40,86,87,88,89,90,93].

## 3. Results and Discussion

### 3.1. Electrodes for ALK

ALK is one of the most mature and industrially implemented hydrogen production technologies and plays a key role in the transition toward a green hydrogen economy [13,14,15,16,17,18,22,29,30]. Its technological relevance is further emphasized in global hydrogen transition roadmaps and market analyses [86,87,88,89,90,91,92,93,94]. ALK offers relatively low infrastructure costs and flexibility in electrode material selection compared with PEM systems [13,14,25,45,46]. However, achieving high electrocatalytic activity, long-term durability, and corrosion resistance under strongly alkaline conditions remains a major scientific and technological challenge [42,43,44,45,96,97,98,99].

The HER at the cathode determines overall system efficiency. Although platinum-based materials exhibit near-zero overpotential and excellent kinetics, their scarcity and high cost limit large-scale deployment [25,42,43]. Consequently, significant efforts focus on developing non-noble metal catalysts and transition-metal-based systems [42,43,44,100,101,102]. Nickel remains the most widely used cathode material in ALK electrolysis due to its good electrical conductivity, corrosion resistance, and intrinsic catalytic activity in alkaline media [25,43,45]. Alloying nickel with molybdenum, iron, or chromium enhances HER kinetics and improves structural stability under industrial conditions [42,43,44,97,103,104,105].

Recent studies highlight the importance of transition metal hydroxides (M–OH) and heterostructured systems (X/M–OH; X = metal, oxide, chalcogenide, phosphide) as highly promising HER catalysts in alkaline media [44,98,99,104,105,106,107,108,109,110,111,112]. These materials promote water dissociation—the rate-determining step in alkaline HER—while improving charge transfer characteristics compared to pure hydroxides [42,43,44,98,105,110]. In alkaline media, the rate-determining step depends on the reaction pathway and catalyst structure. The HER is often limited by water dissociation (Volmer step), particularly on transition-metal surfaces, where H–OH bond cleavage is kinetically demanding [42,43,44,45,98]. In contrast, the OER is intrinsically slower due to its complex four-electron transfer mechanism and multiple intermediate steps [42,47,48]. Therefore, both HER and OER can act as limiting processes depending on catalyst composition, electrode structure, and operating conditions. While HER limitations are typically associated with water activation kinetics, OER limitations arise from sluggish reaction pathways and high overpotentials. This distinction explains the different design strategies used for cathode and anode catalysts in ALK electrolysis. Rational heterostructure engineering (doping, core–shell architectures, multiphase slabs, strain-induced interfaces) enables enhanced catalytic activity and long-term stability [44,98,108,112]. Mechanistic understanding of interfacial effects and electronic structure modulation is therefore crucial for further catalyst optimization [42,43,44,45,98,100,113].

**Figure 7:**
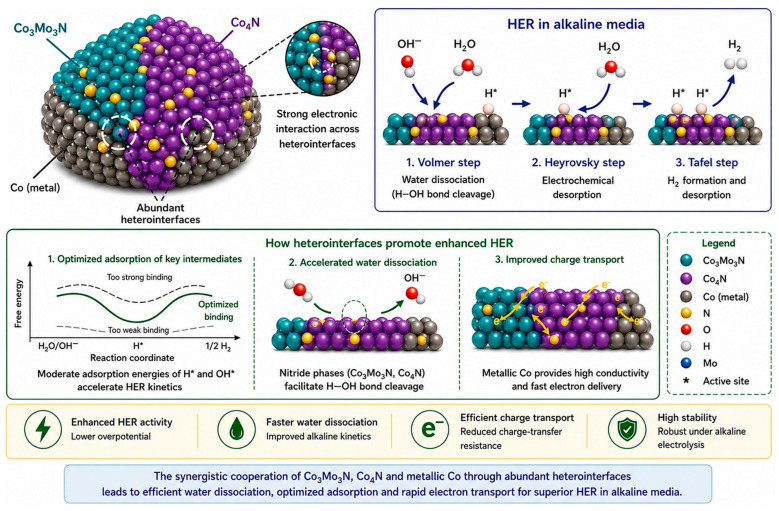
Author’s schematic summary of the structural and electrocatalytic performance of the Co_3_Mo_3_N/Co_4_N/Co heterostructure catalyst for the alkaline (ALK) hydrogen evolution reaction (HER).

To further illustrate the role of heterostructure engineering in enhancing alkaline HER activity, a representative example of a transition-metal nitride catalyst is summarized in Figure 7. The scheme presents the structural concept and catalytic behavior of a Co_3_Mo_3_N/Co_4_N/Co heterostructure, highlighting the synergistic interactions between the individual phases that contribute to improved electrocatalytic performance.

Figure 7 illustrates a multiphase architecture composed of Co_3_Mo_3_N and Co_4_N nitrides integrated with metallic cobalt domains, forming abundant heterointerfaces within the catalyst structure. Such interfaces play a key role in modulating the electronic properties of active sites and optimizing the adsorption energies of reaction intermediates involved in the HER process. In alkaline media, where water dissociation represents a critical kinetic step, the presence of transition-metal nitride phases facilitates the cleavage of the H–OH bond, while metallic cobalt improves electrical conductivity and promotes efficient charge transfer.

Overall, the schematic highlights how rational heterostructure design can enhance catalytic activity by combining complementary functionalities of different phases, including improved water dissociation kinetics, optimized hydrogen adsorption, and efficient electron transport. These features collectively contribute to enhanced HER performance and stability under ALK electrolysis conditions, demonstrating the potential of multiphase nitride systems as promising non-noble metal catalysts for ALK water electrolysis [112].

The OER at the anode is kinetically more demanding and typically requires optimized transition metal oxides or noble metal-based catalysts [42,47]. Common industrial anode materials include nickel, stainless steel, and iron-based substrates, whereas dimensionally stable anodes (DSAs) coated with IrO_2_ or RuO_2_ offer improved activity and durability in harsh alkaline environments [45,47,96]. Nickel-based layered double hydroxides and NiFe systems are widely investigated due to their favorable OER kinetics and structural adaptability [47,48]. The development of robust anode materials remains essential for extending electrolyzer lifetime and improving energy efficiency under high current densities [42,45,97].

From a systems perspective, ALK technology must also be evaluated in the context of grid integration, renewable coupling, and techno-economic optimization [33,34,35,36,37,38,39,94]. Market expansion and policy frameworks further accelerate deployment [86,87,88,89,90,91,92,93]. Although alternative technologies such as PEM electrolysis [49,50,51,52,53,54,55,56,57,58] and SOEC cells [59,60,61,62,63,64,65,66,67,68,69,70,71,72,73] offer specific advantages, ALK electrolysis remains highly competitive due to material availability and industrial scalability [13,45,74,75,76,77].

Recent progress in AEM electrolyzers attempts to combine the advantages of ALK and PEM systems, with extensive research on membranes, catalyst durability, and operational stability [74,75,76,77,78,79,80,81,82,83,84,85]. Comparative analyses of electrolyzer configurations highlight the importance of electrode design, membrane conductivity, and degradation mechanisms for future hydrogen infrastructure development [51,52,53,74,83,84].

Overall, the advancement of electrode materials for ALK electrolysis requires an integrated approach combining catalyst design, interfacial engineering, durability assessment, and system-level optimization [42,43,44,45,97,98,99]. For industrial-scale hydrogen production, electrocatalysts must meet specific performance targets. Typical requirements include current densities exceeding 200–500 mA cm^−2^, operational lifetimes above 10,000 h, and high energy efficiency corresponding to low cell voltages [33,39,86,87,88,89,90]. In addition, reducing noble metal loading is critical for economic viability, particularly in PEM systems where Ir and Pt remain dominant [11,12,33,41]. From a system perspective, overall energy consumption (kWh kg^−1^ H_2_) and material cost per unit hydrogen production are key parameters determining large-scale applicability. These considerations highlight the importance of balancing catalytic activity, durability, and cost in the development of next-generation electrode materials.

To enable a systematic comparison of catalytic performance across different material systems, key electrochemical parameters reported in the literature are summarized in Table 5. These include overpotential at defined current densities, Tafel slopes, and stability test durations, which are critical indicators of catalytic activity, reaction kinetics, and long-term durability [100,101,102,103,104,105,106,107,108,109,110,111,112]. The tabulated data provide a quantitative basis for evaluating the performance of transition-metal-based catalysts relative to noble-metal systems and highlight recent progress toward achieving high current densities with reduced material cost [100,102,106,112].

For the HER process, several catalysts exhibit extremely low overpotentials at the benchmark current density of 10 mA cm^−2^. For instance, NiO@MoO_3−*x*_/Ni demonstrates an overpotential as low as 7 mV, indicating highly favorable hydrogen adsorption/desorption kinetics and efficient charge transfer [106]. Similarly, the PdPtRhRuAu high-entropy alloy aerogel (HEAA) shows a very low HER overpotential of 12 mV with a small Tafel slope of 17 mV dec^−1^, reflecting rapid reaction kinetics typical of noble-metal-based catalytic systems [100]. Another example is Ru–O–Mo (Ru/MoO_2_), which requires only 16 mV to achieve 10 mA cm^−2^ in alkaline electrolyte [103].

Several catalysts demonstrate remarkable activity at significantly higher current densities, which is critical for industrial electrolysis. The MoNi_4_/MoO_2_@Ni catalyst achieves 500 mA cm^−2^ at 65 mV, highlighting the strong synergistic effect between nickel and molybdenum active sites [102]. Even higher current densities are obtained with Pt/Ni–Mo, which reaches 2000 mA cm^−2^ at 113 mV in alkaline saline electrolyte while maintaining stable operation for 140 h at temperatures up to 80 °C [102]. These results demonstrate the potential of hybrid Pt–Ni–Mo architectures for high-current-density electrolysis systems.

Transition-metal nitrides, phosphides, and oxide-based catalysts also show promising catalytic activity as cost-effective alternatives to noble metals. For example, Co_3_Mo_3_N/Co_4_N/Co exhibits balanced HER and OER activity, enabling overall water splitting at a cell voltage of 1.58 V with stable operation for 100 h at 200 mA cm^−2^, maintaining nearly 100% current retention [112]. Likewise, CoFe_2_O_4_@Ni_2_P/NF demonstrates bifunctional catalytic behavior, achieving overall water splitting at 1.60 V in alkaline electrolyte [105].

For the OER, catalysts such as Fe hydroxide@CoS and NiFe@NiCr-LDH show overpotentials of approximately 270 mV at 10 mA cm^−2^, which is typical for transition-metal-based OER catalysts in alkaline media [107,113]. These catalysts generally exhibit good durability due to the formation of active oxyhydroxide species under operating conditions.

Stability remains one of the most important parameters for practical hydrogen production technologies. Several catalysts demonstrate long operational lifetimes, including FeIr/NF, which operates for 504 h at 150 mA cm^−2^ [102]. Similarly, Pt cluster-NiCoP@NF nanowires retain catalytic activity after 5000 electrochemical cycles and maintain stable performance during 30 h operation at 500 mA cm^−2^ [111]. Such durability is essential for the development of practical ALK electrolyzers.

Overall, the data presented in Table 5 highlight the significant progress achieved in the design of highly active and durable electrocatalysts for alkaline water splitting. Recent advances focus on multi-component catalysts, defect engineering, and heterostructure interfaces, which enhance catalytic activity through electronic structure modulation and synergistic effects between active phases.

**Figure 8:**
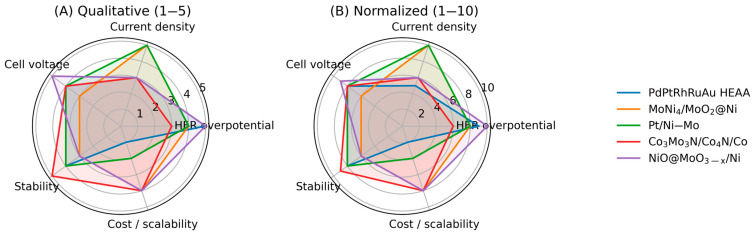
Comparative performance of electrocatalysts for alkaline (ALK) water electrolysis. (**A**) Qualitative comparison on a scale from 1 (poor) to 5 (excellent). (**B**) Normalized comparison on a scale from 2 (poor) to 10 (excellent). The evaluated parameters include catalytic activity (HER overpotential at 10 mA cm^−2^), achievable current density, energy efficiency, operational stability, and cost/scalability. The scoring system is based on normalized values derived from representative literature data [100,102,106,112]. Stability categories correspond to approximate operational ranges (short-term < 50 h, medium-term 50–200 h, long-term > 200 h), while cost/scalability reflects both material composition (e.g., noble metal content) and synthesis complexity. The radar plots provide a semi-quantitative comparison of performance trade-offs and are intended for comparative visualization rather than absolute quantitative assessment.

To provide an intuitive comparison of the catalytic performance metrics summarized in Table 5, a radar-plot analysis of five representative electrocatalysts is presented in Figure 8. The selected catalysts include PdPtRhRuAu HEAA [100], MoNi_4_/MoO_2_@Ni [102], Pt/Ni–Mo [102], Co_3_Mo_3_N/Co_4_N/Co [112], and NiO@MoO_3−*x*_/Ni [106], representing both noble-metal-based catalysts and earth-abundant transition-metal systems. Figure 8A presents a qualitative comparison using a 1–5 scale, where HER overpotential, achievable current density, overall cell voltage, operational stability, and cost/scalability are evaluated. Figure 8B shows a normalized quantitative comparison (1–10 scale), illustrating the relative performance trends and directionality of the evaluated parameters.

The radar plots reveal several key trends. Noble-metal-containing catalysts, such as PdPtRhRuAu HEAA [100] and Pt/Ni–Mo [102], exhibit excellent catalytic activity characterized by low HER overpotentials and favorable reaction kinetics. However, these systems score lower in the cost/scalability category due to the use of precious metals.

In contrast, transition-metal-based catalysts, such as MoNi_4_/MoO_2_@Ni [102] and Co_3_Mo_3_N/Co_4_N/Co [112], demonstrate competitive catalytic performance at higher current densities while offering significantly improved economic feasibility and scalability. These materials benefit from synergistic interactions between metal centers and support structures, which enhance catalytic activity and stability.

The NiO@MoO_3−*x*_/Ni catalyst [106] stands out due to its extremely low HER overpotential, which is attributed to oxygen-vacancy-rich MoO_3−x_ structures that modify the electronic environment of active sites and optimize hydrogen adsorption energies.

The radar-plot comparison highlights the activity–stability–cost trade-off between noble-metal-based and transition-metal-based catalysts. While noble metals still provide the highest intrinsic catalytic activity, transition-metal systems offer more promising prospects for large-scale hydrogen production due to their lower cost and higher material abundance.

High-entropy alloys (HEAs) and high-entropy oxides (HEOs) have emerged as promising catalyst platforms due to their tunable electronic structures and multi-element synergistic effects [100,101,102]. These materials often exhibit catalytic activity comparable to noble-metal-based systems, particularly in HER and OER reactions [100,102]. However, their practical implementation must be evaluated in terms of cost–performance trade-offs.

Although HEAs/HEOs reduce reliance on critical raw materials by partially replacing noble metals, their synthesis often involves complex multi-step processes, high-purity precursors, and limited scalability [100,101,102]. Consequently, the overall cost advantage over conventional noble-metal catalysts is not always straightforward [11,12,33,41]. From a performance perspective, HEAs/HEOs offer high catalytic activity and structural stability, but challenges remain in achieving reproducible large-scale fabrication and long-term durability under industrial conditions [33,41].

Overall, recent advances in catalyst design demonstrate that transition-metal-based heterostructures and defect-engineered materials can approach the catalytic activity of noble metals while maintaining superior cost efficiency and scalability, making them promising candidates for large-scale ALK water electrolysis.

### 3.2. Electrodes for PEM Electrolysis

PEM electrolyzers use different electrode materials compared to ALK due to the different operating conditions, particularly the acidic environment created by the polymer electrolyte membrane [13,25,49,51,53,55,56,58,114,115,116,117,118,119,120,121,122,123,124]. The electrode materials in PEM electrolyzers must be corrosion-resistant in acidic conditions and catalytically active for the HER and OER [25,49,51,53,55,56,58,59]. Common materials used for the electrodes in PEM electrolyzers include platinum and PGMs [25,51,53,55,56,58,59,60]. Platinum is often used as a catalyst for the cathode in PEM electrolyzers because of its high activity for the HER [25,51,53,55,56,58]. Other platinum group metals, such as iridium and ruthenium, are used for the anode due to their catalytic activity for the OER and their stability in acidic environments [25,51,53,54,55,56,59]. To reduce the cost, platinum is sometimes alloyed with other metals to improve its performance and reduce the amount of platinum required [25,55,56,58,59,60]. These platinum alloy catalysts can provide a balance between cost and catalytic activity [25,55,56,58]. Iridium(IV) oxide and ruthenium(IV) oxide are also commonly used as catalysts for the anode in PEM electrolyzers because of their excellent OER activity and stability in acidic media [25,51,53,54,55,56,58]. They are particularly effective for the OER, which is typically the more challenging half-reaction in terms of overpotential [25,51,53,55].

In PEM electrolyzers, precious metal catalysts are typically supported on high-specific-surface-area carbon materials, which enhance catalyst dispersion, improve electrical conductivity, and reduce the required noble metal loading [25,49,51,53,55,56,58]. Carbon-based supports are widely employed in electrocatalysis due to their favorable physicochemical properties. In PEM and AEM systems, they serve as effective supports for both noble-metal and transition-metal catalysts, enabling improved utilization of active sites and reduced noble metal loading [49,51,53,55]. In ALK electrolysis systems, carbon supports are also applied; however, their long-term stability may be limited under highly oxidative operating conditions, particularly at the anode [42,43,44]. In contrast, carbon-based materials are generally not suitable for high-temperature SOEC systems due to their thermal instability at elevated operating temperatures (>700 °C), where ceramic and oxide-based materials are preferred [59,60,61,62,63,64,65].

Moreover, titanium is sometimes used as a substrate material for the anode due to its corrosion resistance in acidic conditions [25,49,51,53,55]. The catalytic layer, such as IrO_2_ or RuO_2_, is then deposited onto the titanium substrate [25,49,51,53,55,56]. Research is ongoing to find alternative materials that can replace or reduce the use of precious metals in PEM electrolyzers, as these metals are expensive and contribute significantly to the overall cost of the electrolyzer [25,49,51,52,53,55,56,58,59]. Non-precious metal catalysts, conductive metal oxides, and other novel materials are being explored to achieve high catalytic activity and stability at a lower cost [25,49,52,55,56,59,60].

Recently, the use of electrochemical impedance spectroscopy (EIS) method to analyze membrane electrode assemblies (MEAs) in PEM electrolyzers was investigated [25,49,50,52,55]. The MEAs are composed of a short-side chain perfluorosulfonic acid membrane (Aquivion^®^, Solvay, Brussels, Belgium) and an advanced Ir-Ru oxide anode electrocatalyst with varying cathode and anode noble metal loadings [25,49,50,52,55,56]. The research aimed to identify rate-determining steps and quantify the impact of different phenomena on the efficiency and stability of PEM electrolyzers [25,49,50,52,55,56]. EIS was shown to be a valuable diagnostic tool for separating the contributions of various mechanisms influencing polarization characteristics, such as ohmic and polarization resistance [25,49,50,52,55,56,58]. The study also explored the effects of temperature, catalyst loading, and operating potential on the performance and degradation of MEAs [25,49,50,52,55,56]. Key findings include the ability to achieve high electrolysis current densities with low PGM catalyst loading while maintaining high conversion efficiency and stability [25,49,50,52,55,56]. The study also highlighted the importance of the anode process, particularly at low temperatures and potentials, and the significant reduction in polarization resistance with increased temperature, especially for the anode reaction [25,49,50,52,55,56]. The authors concluded that EIS can provide critical insights into the electrode processes contributing to the polarization behavior of PEM electrolysis cells, aiding in the identification of components most affected by degradation [25,49,50,52,55,56]. This non-invasive technique can guide research efforts towards improving specific cell components and serve as a diagnostic tool for the state of the electrolysis system [25,49,50,52,55,56,58].

**Figure 9:**
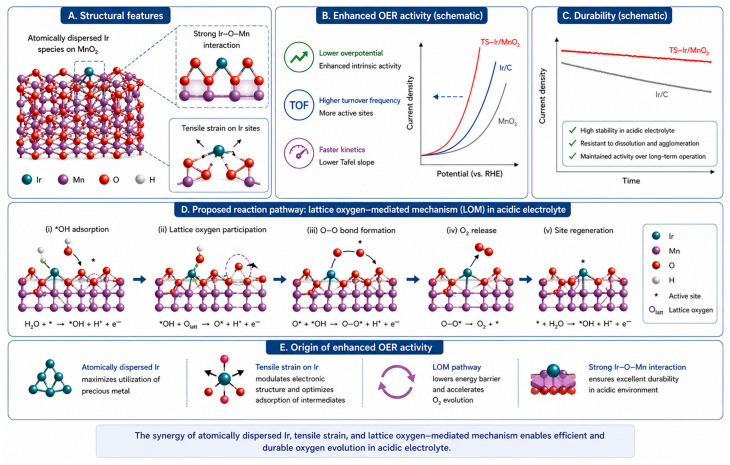
Author’s schematic summary of the TS–Ir/MnO_2_ electrocatalyst (TS—tensile-strained) including structural characterization, oxygen evolution reaction (OER) activity, durability, and the proposed reaction pathway in acidic electrolyte.

Figure 9 presents a schematic summary of the TS–Ir/MnO_2_ electrocatalyst, where TS denotes tensile-strained, including its structural characteristics, OER performance, durability, and the proposed reaction pathway in an acidic electrolyte [114].

The catalyst consists of atomically dispersed Ir species stabilized on a MnO_2_ support, where the interaction with the oxide lattice induces tensile strain in the Ir active sites. This structural configuration modifies the electronic properties of Ir and enhances its catalytic activity toward the OER. Electrochemical measurements indicate that the TS–Ir/MnO_2_ catalyst exhibits high OER performance in acidic media, reaching an overpotential of approximately 198 mV at 10 mA cm^−2^ and a Tafel slope of about 56.6 mV dec^−1^, indicating favorable reaction kinetics. In addition, the catalyst demonstrates high mass activity and good durability during extended operation [114].

As illustrated in Figure 9, the enhanced activity is associated with a localized lattice oxygen–mediated mechanism (LOM), in which lattice oxygen participates in the oxygen evolution process. These results highlight the potential of strain-engineered, atomically dispersed Ir catalysts for improving the efficiency and stability of anode materials in PEM water electrolyzers.

The important research challenges include understanding the levels of impurities that real PEM electrolyzers are likely to encounter, as even trace amounts can be significant over the long operating lifetime of the system [25,49,50,51,52,53,54,55,56,115]. There is also a lack of information on the solubility of species originating from balance-of-plant (BoP) components (e.g., piping, seals, and auxiliary system materials) in ultra-pure water, as well as on the actual composition of deionized feedwater in commercial-scale systems. These species may include both common ionic impurities (e.g., Na^+^, K^+^, Cl^−^) and elements leached from system materials. More research is also needed under industrial conditions, especially with regard to anions and organic compounds, to assess their impact on PEM electrolyzer performance [25,49,50,51,52,53,54,55,56,115]. The potential impact of more active catalysts with lower loadings on susceptibility to poisoning and dissolution is also a challenge [25,49,50,51,52,53,54,55,56,115], as well as the effects of organic impurities, which are largely unexplored in the literature [25,49,50,51,52,53,54,55,56,115]. Current challenges also include the economic benefits of using lower quality water and the potential of organic water electrolysis technology, and the impact of solid and microbial impurities on PEM electrolyzer performance, which has not yet been assessed [25,49,50,51,52,53,54,55,56,115]. Addressing these challenges is crucial for the development of robust and efficient PEM electrolyzer systems that can operate with a variety of water sources and maintain high performance over extended periods [25,49,50,51,52,53,54,55,56,115].

Table 6 summarizes selected high-performance electrocatalysts reported for the HER, OER, and overall water splitting in systems relevant to PEM electrolysis. The presented materials demonstrate significant improvements in catalytic activity, reaction kinetics, and operational stability compared with conventional benchmark catalysts such as IrO_2_ and Pt-based systems.

Platinum-based catalysts remain the benchmark for the HER in PEM electrolysis systems due to their near-zero overpotential and fast reaction kinetics. As shown in Table 6, a Pt/C catalyst (0.3 mg_Pt_ cm^−2^) achieves a current density of 1000 mA cm^−2^ at an overpotential of only ~5 mV, with a low Tafel slope of approximately 30 mV dec^−1^, indicating highly efficient HER performance under acidic conditions [119]. However, despite their outstanding activity and stability, the large-scale application of Pt-based catalysts is limited by their high cost and scarcity. Therefore, significant research efforts are focused on developing alternative catalysts that can approach the performance of Pt while reducing noble metal loading.

Among the OER catalysts operating in acidic electrolytes, the TS–Ir/MnO_2_ catalyst shows excellent activity, achieving 10 mA cm^−2^ at an overpotential of 198 mV with a Tafel slope of 56.6 mV dec^−1^ and long-term stability for 200 h at 500 mA cm^−2^ in 0.1 M HClO_4_ [114]. The high performance is attributed to atomically dispersed, tensile-strained Ir active sites that enhance intrinsic catalytic activity while maintaining stability in strongly acidic environments.

Another highly active system is the RuIr-NC catalyst composed of Ru–Ir nanosheets, which demonstrates excellent bifunctional performance for both HER and OER in 0.5 M H_2_SO_4_ [116]. For the HER, the catalyst achieves 10 mA cm^−2^ at an overpotential of only 46 mV with a Tafel slope of 32 mV dec^−1^, indicating fast reaction kinetics [116]. For the OER, the same material exhibits an overpotential of 165 mV at 10 mA cm^−2^ and a Tafel slope of 40 mV dec^−1^, together with operational stability exceeding 120 h [116]. When used as both cathode and anode in a full electrolyzer configuration (RuIr-NC‖RuIr-NC), the system reaches 10 mA cm^−2^ at a cell voltage of 1.485 V, demonstrating efficient overall water splitting under acidic conditions [116].

Perovskite-based catalysts are also being investigated as promising alternatives to conventional IrO_2_ catalysts. The double perovskite Sr_2_CaIrO_6_ exhibits good OER activity with an overpotential of 250 mV at 10 mA cm^−2^ in 0.1 M HClO_4_ and demonstrates remarkable durability in a PEM water electrolyzer, maintaining stable operation for 450 h at 2 A cm^−2^ [117]. Such materials offer improved structural stability and potentially more efficient utilization of iridium.

Another highly active OER catalyst is Ru_6_W_4_O_x_-400 °C, which achieves an overpotential of 140 mV at 10 mA cm^−2^ and a Tafel slope of 51.4 mV dec^−1^ in acidic electrolyte, while maintaining stable operation for approximately 150 h [118]. The incorporation of tungsten into the Ru-based oxide structure is believed to improve both catalytic activity and structural stability.

Recent studies also focus on reducing the loading of precious metals in practical PEM electrolyzer systems. A low-iridium catalyst applied in a 10-cell industrial PEM stack demonstrated a 30-fold increase in mass activity compared with IrO_2_, while maintaining stability during 3700 h of cycling at an iridium loading of only 0.25 mg_Ir_ cm^−2^ [119]. Similarly, the IrO_x_/Ti_4_O_7_ catalyst layer used in power-to-X (P2X) systems achieved approximately nine-fold higher mass activity than IrO_2_, remaining stable for more than 50 h at 1 A cm^−2^ in a PEM membrane electrode assembly configuration [120].

Other catalyst architectures include core–shell IrCo@IrCoO_x_ nanoparticles, which exhibit OER activity with an overpotential of 259 mV at 10 mA cm^−2^ and a Tafel slope of 59 mV dec^−1^, maintaining stable operation for 55 h at 50 mA cm^−2^ under acidic conditions [121]. Structural engineering in such core–shell systems allows tuning of electronic properties and improved catalytic efficiency.

In addition to noble-metal-based catalysts, emerging materials such as atomically dispersed Ru on NiCo LDH demonstrate promising bifunctional performance for overall water splitting, reaching approximately 1.45 V at 10 mA cm^−2^ in model systems [122]. Furthermore, MXene-derived MBenes (transition-metal borides) and transition-metal sulfides or phosphides represent broader classes of catalysts capable of catalyzing both HER and OER across a wide pH range, often achieving 10 mA cm^−2^ at overpotentials between 20 and 150 mV depending on composition and operating conditions [123,124].

Overall, the data summarized in Table 6 highlight several key trends in the development of advanced electrocatalysts for PEM electrolysis. These include strain engineering, atomic dispersion of active metals, alloying strategies, core–shell architectures, and the use of conductive supports, all aimed at improving catalytic activity while reducing the amount of expensive noble metals required. Continued research in this area is essential for developing cost-effective and durable catalysts suitable for large-scale hydrogen production using PEM electrolyzers.

**Figure 10:**
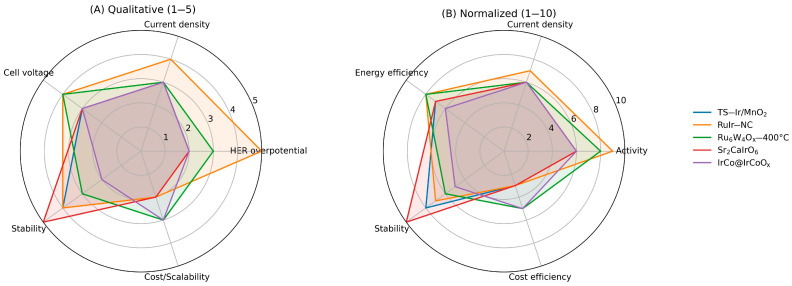
Comparative performance of electrocatalysts for proton exchange membrane (PEM) water electrolysis. (**A**) Qualitative comparison on a scale from 1 (poor) to 5 (excellent). (**B**) Normalized comparison on a scale from 2 (poor) to 10 (excellent). The evaluated parameters include catalytic activity (HER/OER overpotential at 10 mA cm^−2^), achievable current density, energy efficiency (based on reported full-cell voltage where available), operational stability, and cost/scalability. Scores are derived from normalized values reported in representative studies [114,116,117,118,121]. Activity values represent either HER or OER performance depending on the reported reaction for each catalyst. Stability categories correspond to approximate operational ranges (<50 h, 50–200 h, >200 h), while cost/scalability reflects both material composition and synthesis complexity. The radar plots provide a semi-quantitative comparison of performance trade-offs and are intended for comparative visualization rather than absolute quantitative assessment.

Figure 10 compares the performance of representative electrocatalysts used in PEM water electrolysis using radar plots that highlight the interplay between catalytic activity, stability, and economic feasibility. Figure 10 (Panel A) presents a qualitative comparison (1–5 scale) of key performance indicators, including HER overpotential, achievable current density, overall cell voltage, operational stability, and cost/scalability. Among the analyzed systems, the RuIr-NC nanosheet catalyst exhibits the most balanced performance profile, particularly in terms of low HER overpotential and high catalytic activity in acidic media, consistent with previously reported values of ~46 mV at 10 mA cm^−2^ for HER and ~165 mV for OER in 0.5 M H_2_SO_4_ [116]. Noble-metal-based catalysts generally demonstrate superior electrochemical performance; however, their scalability is limited by the high cost and scarcity of iridium and ruthenium. In contrast, catalysts such as TS–Ir/MnO_2_ and Sr_2_CaIrO_6_ show excellent stability under acidic conditions, with long-term operation exceeding 200 h and up to 450 h in PEMWE configurations, respectively, highlighting the importance of robust oxide frameworks for sustained OER activity [114,117].

Figure 10 (Panel B) presents a normalized quantitative comparison (1–10 scale) that further emphasizes the performance directionality of the analyzed catalysts. In this representation, activity, current density capability, energy efficiency, stability, and cost efficiency are scaled to allow a more direct comparison of catalytic systems with different compositions and operating metrics. The RuIr-NC catalyst again demonstrates the highest activity and energy efficiency due to its ultrathin nanosheet morphology and synergistic Ru–Ir electronic structure, which enhances reaction kinetics in acidic environments [116]. Meanwhile, materials such as Ru_6_W_4_O_x_ and IrCo@IrCoO_x_ represent strategies aimed at reducing noble metal loading while maintaining acceptable catalytic performance, resulting in improved cost efficiency compared with conventional IrO_2_-based catalysts [118,121]. Perovskite-derived structures, such as Sr_2_CaIrO_6_, stand out in terms of long-term stability, demonstrating that crystal-structure engineering can significantly improve catalyst durability under highly oxidative PEM electrolysis conditions [117].

The comparison highlights a clear activity–stability–cost trade-off between noble-metal-rich catalysts and emerging mixed-metal or transition-metal-based systems. While noble-metal catalysts currently provide the highest catalytic activity and efficiency in acidic PEM electrolysis environments, the development of materials with reduced noble metal content—such as alloyed oxides, core–shell architectures, or atomically dispersed active sites—represents a key strategy for improving the economic viability of large-scale hydrogen production technologies [118,121,122,123,124].

Overall, recent advances in PEM electrolyzer electrode design demonstrate that atomically dispersed, strain-engineered, and mixed-metal catalysts—combined with conductive supports and core–shell architectures—can achieve high catalytic activity and stability in acidic environments while reducing noble-metal loading, making them promising candidates for cost-effective and durable large-scale PEM water electrolysis.

### 3.3. Electrodes for SOEC

The high-temperature environment and the need for materials that are stable under oxidizing and reducing conditions on the anode and cathode sides, respectively, dictate the choice of electrode materials for SOEC. On the cathode side, where hydrogen is produced, a ceramic-metal composite (cermet) of nickel and YSZ is often used [65,69,125,126,127,128]. Nickel acts as the catalyst for the HER and provides electronic conductivity, while the YSZ phase ensures ionic conductivity for the transport of oxygen ions. On the anode side, where oxygen is evolved, materials such as LSM or lanthanum strontium ferrite (LSF) are used [59,69,125]. These perovskite oxides are stable in the oxidizing environment and exhibit mixed ionic and electronic conductivity, which is necessary for the OER.

For intermediate-temperature SOEC at 500–700 °C, materials with higher ionic conductivity, such as lanthanum strontium cobaltite (LSC) or LSCF, are applied [66,69,126]. These materials have better catalytic activity and ionic conductivity at lower temperatures compared to LSM.

As an alternative to YSZ, electrolytes made of scandia-stabilized zirconia (ScSZ) or GDC can be used in conjunction with the electrode materials mentioned above [60,66,127]. These electrolytes offer higher ionic conductivity and better performance at lower temperatures.

The choice of electrode materials in SOEs is critical for achieving high efficiency and durability. The materials must withstand the thermal cycling, resist chemical degradation, and maintain their catalytic activity and electrical conductivity over the lifetime of the electrolyzer [32,68,69,128]. Ongoing research aims to develop new materials and optimize existing ones to improve the performance and reduce the cost of SOEs [125,126,127,128].

The electrochemical performance of oxygen electrodes in SOEC systems is strongly influenced not only by the intrinsic catalytic activity of the electrode materials but also by their microstructural architecture. In particular, parameters such as porosity, phase connectivity, particle size distribution, and the extent of TPB regions play a key role in determining the efficiency of the OER and the transport of ions and electrons within the electrode [59,66,69].

**Figure 11:**
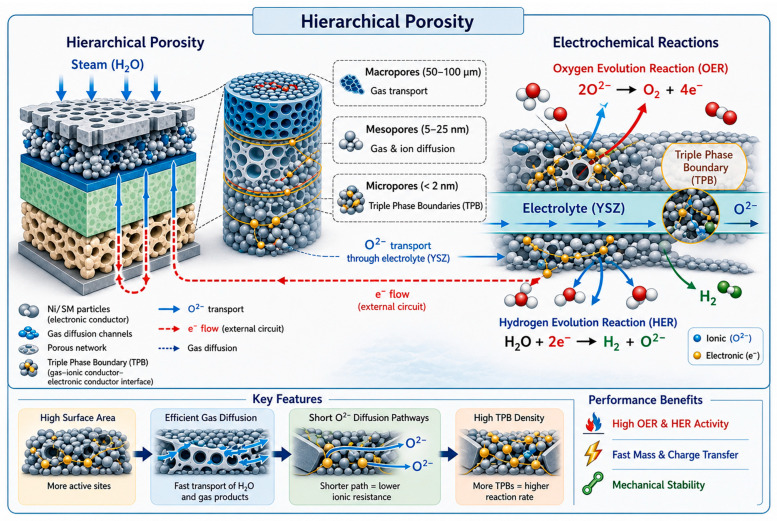
Author’s schematic illustration of hierarchical porosity in a solid oxide electrolysis cell (SOEC) electrode and its impact on electrochemical performance. Steam (H_2_O) diffuses through the hierarchical porous structure toward the triple phase boundary (TPB), where the hydrogen evolution reaction (H_2_O + 2e^−^ → H_2_ + O^2−^) takes place. The generated oxygen ions (O^2−^) are transported through the yttria-stabilized zirconia (YSZ) electrolyte to the oxygen electrode, where the oxygen evolution reaction (2O^2−^ → O_2_ + 4e^−^) occurs. The electron flow (e^−^) is indicated separately as transport through the external circuit, while ionic transport (O^2−^) is shown through the electrolyte. Hierarchical porosity (macro-, meso-, and micropores) enhances gas diffusion, increases TPB density, and shortens O^2−^ diffusion pathways, resulting in improved electrochemical performance. SM stands for support material.

Figure 11 provides a schematic overview of the structural and electrochemical characteristics of high-performance oxygen electrodes used in SOEC.

The figure highlights the importance of a hierarchical porous structure, consisting of macro-, meso-, and micropores, which enables efficient gas transport and provides a large active surface area for electrochemical reactions. Such multiscale porosity facilitates the diffusion of oxygen species and steam, while maintaining continuous pathways for both electronic and ionic conduction.

The schematic also illustrates the role of interconnected MIEC phases, which extend the electrochemically active regions beyond the conventional TPBs, thereby enhancing the overall reaction kinetics. In addition, the fundamental electrochemical processes, including the OER and HER, as well as the associated transport pathways of ions, electrons, and gaseous species, are depicted.

Another important aspect emphasized in Figure 11 is the influence of microstructural optimization on electrode durability and stability. A homogeneous distribution of catalytic phases and controlled particle morphology helps to mitigate degradation processes such as coarsening, phase segregation, or loss of active surface area during long-term high-temperature operation. Recent studies have demonstrated that careful control of electrode architecture can significantly improve charge transport pathways and reaction rates, ultimately leading to higher current densities and improved SOEC efficiency [125].

These insights highlight that, in addition to the development of new electrode compositions, microstructural engineering represents a critical strategy for improving the performance and long-term stability of oxygen electrodes in solid oxide electrolysis systems.

Table 7 summarizes selected high-performance electrocatalysts used in SOEC for both the HER and the OER. The table compares representative electrode materials in terms of operating electrolyte, achievable current density, Tafel slope, and long-term stability under high-temperature electrolysis conditions.

For the fuel electrode (HER side), nickel-based cermets remain the most widely used materials due to their excellent catalytic activity for steam reduction and high electronic conductivity. In particular, Ni–YSZ cermets demonstrate stable operation at high temperatures, reaching current densities of approximately 1000 mA cm^−2^ at a cell voltage of about 1.30 V at 800 °C with durability exceeding 1000 h [65,69]. Similar performance is observed under co-electrolysis conditions, where Ni–YSZ electrodes maintain comparable current densities and stability, confirming their suitability for combined H_2_O/CO_2_ electrolysis processes [62,65]. Alternative composites such as Ni–GDC and Ni–ScSZ offer improved ionic conductivity due to the properties of the ceria- or scandia-based electrolytes, enabling high current densities at slightly lower operating temperatures [60,66,69]. More advanced fuel electrodes based on perovskite materials, such as LSFN or LSCM–GDC composites, have also been investigated. These materials can achieve very high current densities, exceeding 1500 mA cm^−2^ or even above 2000 mA cm^−2^ under certain operating conditions, although their long-term stability remains a challenge [127,128].

On the oxygen electrode side (OER), perovskite-type oxides dominate due to their mixed ionic–electronic conductivity and chemical stability in oxidizing environments. Classical materials such as LSM exhibit good durability, operating at current densities around 500 mA cm^−2^ with stability exceeding 1000 h [59,69]. However, more advanced compositions such as LSCF or LSC provide improved catalytic activity and lower Tafel slopes, allowing current densities approaching 800–1000 mA cm^−2^ at similar voltages [66,69]. Composite structures, such as LSM–YSZ or LSCF–GDC electrodes, further enhance electrochemical performance by improving ionic transport and expanding the active reaction zone [69,125].

Recent studies have also explored novel electrode architectures and catalytic enhancements. For example, Ru-infiltrated LSCF electrodes exhibit improved OER activity due to the catalytic properties of ruthenium, enabling stable operation at elevated current densities [126]. Layered Ruddlesden–Popper structures, such as Pr_2_Ni_0.8_Co_0.2_O_4_+δ, also demonstrate promising performance, combining high catalytic activity with good structural stability at intermediate temperatures [69]. In addition, newly developed perovskite compositions such as Ba_0.95_LaFeO_3_−δ have demonstrated extremely high current densities exceeding 3000 mA cm^−2^ under high-temperature operation, highlighting the potential of advanced oxide materials for next-generation SOEC systems [128].

Overall, the data presented in Table 7 indicate that nickel-based cermets remain the benchmark materials for the hydrogen electrode, while perovskite-type mixed ionic–electronic conductors dominate the oxygen electrode side. Recent research trends focus on microstructural optimization, composite electrode architectures, and catalytic infiltration strategies to improve both electrochemical performance and long-term durability. Continued development of advanced oxide materials and electrode designs is expected to further enhance the efficiency and operational stability of SOEC systems.

**Figure 12:**
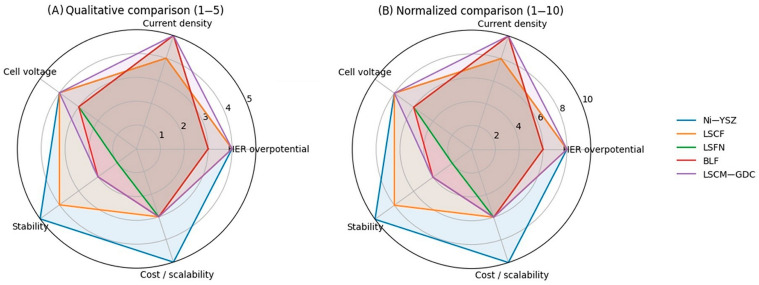
Comparative performance of electrode materials for solid oxide electrolysis cell (SOEC). (**A**) Qualitative comparison on a scale from 1 (poor) to 5 (excellent). (**B**) Normalized comparison on a scale from 2 (poor) to 10 (excellent). The evaluated parameters include electrochemical activity (SOEC performance, based on reported current density and overpotential), achievable current density, energy efficiency, operational stability, and cost/scalability. Scores are derived from normalized values reported in representative studies [65,69,127,128]. Due to the high-temperature operation and different reaction mechanisms in SOEC systems, activity reflects overall electrode performance rather than distinct HER or OER contributions. Stability categories correspond to approximate operational ranges reported in the literature, typically distinguishing short-term (<100 h), intermediate (100–500 h), and long-term (>500 h) performance, reflecting the different operating conditions of SOEC systems. Cost/scalability reflects material composition and processing complexity. The radar plots provide a semi-quantitative comparison of performance trade-offs and are intended for comparative visualization rather than absolute quantitative assessment.

The radar-plot comparison presented in Figure 12 provides a concise overview of the performance trade-offs among representative electrocatalysts used in SOEC. The analyzed materials include both conventional Ni-based cermet cathodes and advanced perovskite-type oxide electrodes, allowing a direct comparison of catalytic activity, stability, and practical applicability in high-temperature steam electrolysis. As summarized in Table 7, Ni–YSZ electrodes typically achieve current densities of approximately 1000 mA cm^−2^ at about 1.30 V and 800 °C, while maintaining operational stability exceeding 1000 h [65,69]. This balanced performance profile, together with relatively low material cost and mature fabrication technology, explains the continued industrial relevance of Ni–YSZ electrodes in commercial SOEC stacks [62,65].

In contrast to Ni-based cermets, perovskite-type oxides are primarily explored as OER catalysts at the oxygen electrode. Among these materials, LSCF demonstrates a favorable combination of catalytic activity and durability. Reported electrochemical performance indicates current densities approaching 1000 mA cm^−2^ at approximately 1.30 V and 750 °C with operational stability close to 800 h [61,69]. The improved activity of LSCF originates from its mixed ionic–electronic conductivity and enhanced oxygen surface exchange kinetics, which facilitate oxygen evolution and transport within the electrode structure.

More recently developed perovskite-derived materials have demonstrated even higher catalytic activity under SOEC conditions. For instance, LSFN (La_0.9_Sr_0.1_Fe_0.9_Nb_0.1_O_3_−δ) has been reported to reach current densities exceeding 1500 mA cm^−2^ at 1.5 V and 850 °C [127]. Similarly, composite electrodes based on LSCM–GDC (La_0.75_Sr_0.25_Cr_0.5_Mn_0.5_O_3_ combined with GDC) exhibit high electrochemical performance, achieving approximately 2360 mA cm^−2^ at 800 °C [128]. The enhanced performance of these materials is generally attributed to improved electronic conductivity, higher catalytic activity of transition-metal cations, and the extension of the electrochemically active region beyond the conventional TPB through the incorporation of MIEC phases.

Among the investigated catalysts, BLF exhibits the highest reported activity, with current densities reaching approximately 3170 mA cm^−2^ at 1.5 V and 850 °C [128]. This exceptional catalytic performance is reflected in the radar-plot comparison by the high score assigned to current density and overall electrochemical activity. However, the operational stability of BLF-based electrodes remains relatively limited, with reported lifetimes of approximately 200 h. This observation highlights a common challenge in the development of highly active perovskite electrocatalysts, where increased catalytic activity is frequently accompanied by reduced structural stability under prolonged high-temperature electrolysis conditions.

The comparative radar analysis illustrates several important trends in SOEC electrocatalyst development. Conventional Ni-based cermets continue to offer the best compromise between stability, cost, and scalability, which explains their continued use in practical SOEC systems [62,65]. Perovskite-type oxides significantly enhance oxygen electrode activity due to their MIEC and favorable oxygen exchange kinetics [61,69]. Meanwhile, recently developed perovskite and composite oxide electrodes can achieve extremely high current densities exceeding 2–3 A cm^−2^ under elevated temperature conditions, although their long-term stability still requires further improvement [127,128]. Consequently, future research should focus on improving the structural stability of highly active oxide electrodes, optimizing electrode microstructures, and enhancing electrode–electrolyte compatibility to enable durable SOEC operation at industrially relevant current densities.

In summary, achieving high-performance SOEC operation relies on the careful selection and engineering of electrode materials, where nickel-based cermets provide robust catalytic activity and conductivity for HER, while perovskite-type mixed ionic–electronic conductors offer stable and efficient OER performance, with microstructural optimization and composite architectures further enhancing overall efficiency and durability.

### 3.4. Electrodes for Anion Exchange Membrane Electrolysis

The electrode materials in AEM electrolyzers must be compatible with the alkaline environment and have good catalytic activity for the HER and OER [48,74,75,76,77,85,129,130,131,132,133,134,135]. Nickel is often used as the cathode material in AEM electrolyzers due to its good catalytic activity for the HER in alkaline conditions [42,98]. Nickel-based alloys, such as nickel-molybdenum or nickel-iron, can also be used to enhance the catalytic performance and stability [42,98]. Iron and its alloys can be used as anode materials in AEM electrolyzers for the OER [107]. Iron-based catalysts are less expensive than precious metal catalysts and can exhibit good OER activity in alkaline media [107]. Cobalt and cobalt-based oxides, such as cobalt oxide (Co_3_O_4_) or cobalt-iron oxides (CoFe_2_O_4_), can be used as OER catalysts in AEM electrolyzers [42,107]. These materials are known for their catalytic activity and stability under alkaline conditions.

In some cases, silver is used as a cathode material due to its high catalytic activity for the HER. However, its high cost limits its widespread use. Carbon supports, such as carbon black or graphene, are sometimes used to improve the conductivity and dispersion of the catalysts [42,85]. These supports can be coated with catalyst materials to create high-surface-area electrodes, which can enhance catalytic efficiency in AEM electrolyzers [129].

Transition metal phosphides, carbides, and nitrides are also electrode materials which have been explored as alternative catalysts for both the HER and OER due to their potential for high activity and stability in alkaline environments [124,130,131].

The development of electrode materials for AEM electrolyzers is an active area of research, with a focus on improving catalytic activity, stability, and reducing costs [74,75,76,77,85]. Researchers are also exploring non-precious metal catalysts and novel materials to optimize the performance of AEM electrolyzers [48,129,130,131].

**Figure 13:**
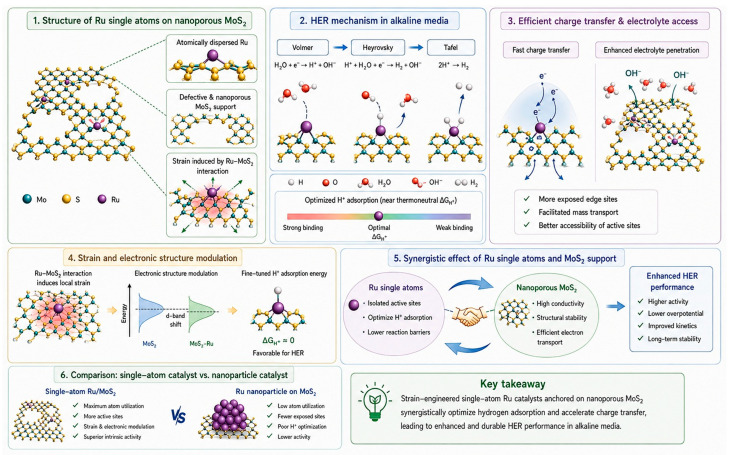
Author’s schematic illustration of strain-engineered Ru single atoms anchored on nanoporous MoS_2_ and their role in optimizing hydrogen adsorption during the HER.

Recent studies demonstrate that single-atom catalysts can significantly improve HER performance in alkaline media [130]. As illustrated in Figure 13, atomically dispersed Ru atoms anchored on nanoporous MoS_2_ serve as highly active catalytic centers and facilitate improved charge transfer at the catalyst–electrolyte interface, leading to enhanced HER activity in alkaline media. The schematic presents the concept of strain-engineered single-atom Ru catalysts, where isolated Ru atoms are stabilized within the defective and nanoporous structure of the MoS_2_ support. This architecture increases the number of exposed edge sites and promotes efficient electrolyte penetration, thereby improving the accessibility of catalytic centers and optimizing hydrogen adsorption during the HER process.

In this system, the interaction between Ru atoms and the MoS_2_ lattice induces local strain and electronic structure modulation, which optimizes the adsorption energy of hydrogen intermediates (H*). As a result, the hydrogen adsorption–desorption process approaches thermoneutral conditions, which is considered ideal for efficient HER catalysis. The presence of isolated Ru atoms also promotes faster charge transfer between the catalyst surface and the electrolyte, reducing kinetic barriers during the reaction [130].

Furthermore, the schematic highlights the synergistic effect between the Ru single atoms and the MoS_2_ support, where the Ru sites serve as the primary catalytic centers while the conductive nanoporous MoS_2_ framework ensures efficient electron transport and structural stability. This cooperative interaction significantly enhances the catalytic activity compared with conventional nanoparticle-based catalysts.

Overall, Figure 13 demonstrates how single-atom catalyst design combined with strain engineering can effectively tune hydrogen adsorption properties and improve HER performance, providing an advanced strategy for developing highly efficient electrocatalysts for alkaline water electrolysis [130].

Table 8 summarizes representative high-performance electrocatalysts reported for AEM systems operating in 1 M KOH electrolyte, highlighting key electrochemical parameters such as overpotential at 10 mA cm^−2^, Tafel slope, and operational stability.

Among the catalysts for the HER, the Ru/np-MoS_2_ single-atom catalyst exhibits the best catalytic performance, requiring an overpotential of only 30 mV to reach 10 mA cm^−2^, together with a low Tafel slope of 31 mV dec^−1^, indicating very fast reaction kinetics and efficient charge transfer at the catalyst surface [130]. Similarly, the RuO_2_/CNT paper electrode shows excellent HER activity with an overpotential of 36 mV at 10 mA cm^−2^, although detailed kinetic and durability parameters were not reported [129].

Transition-metal-based catalysts also demonstrate promising activity while offering improved cost efficiency. The NiMo alloy supported on nickel foam (NiMo/NF) achieves 10 mA cm^−2^ at an overpotential of 60 mV with a Tafel slope of 55 mV dec^−1^ and stable operation exceeding 24 h, confirming its potential as a noble-metal-free HER catalyst [98,110]. The (Fe,Ni)_3_P/NiCoP heterointerface catalyst exhibits similarly low overpotential (52.3 mV at 10 mA cm^−2^) and remarkable durability exceeding 500 h, indicating outstanding long-term stability under alkaline conditions [131]. In contrast, NHCoMX (Ti_3_C_2_ MXene integrated Co-doped Ni(OH)_2_) requires a somewhat higher overpotential of 73 mV with a Tafel slope of 85 mV dec^−1^, but demonstrates multifunctional catalytic activity enabling both HER and OER processes [133]. A less active HER catalyst is NiFeSe/CFP, which requires 186 mV at 10 mA cm^−2^, although it still maintains moderate stability for approximately 20 h [134].

For the OER, Ce-doped NiFe-LDH shows one of the most favorable performances with an overpotential in the range of 240–260 mV at 10 mA cm^−2^ and a Tafel slope of 59 mV dec^−1^, maintaining stable operation for approximately 50 h [113]. In comparison, Ni NPs/GDY exhibits slightly higher overpotential (294 mV) but provides good durability, remaining stable for 90 h at 10 mA cm^−2^, which highlights the stabilizing role of the graphdiyne support [132]. The NHCoMX system also catalyzes OER but requires a higher overpotential of 310 mV, reflecting slower kinetics under alkaline conditions [133].

Several catalysts were also evaluated for overall water splitting performance. The NHCoMX catalyst enables overall electrolysis at a cell voltage of 1.72 V at 10 mA cm^−2^, demonstrating the feasibility of bifunctional electrocatalysis within a single material [133]. An even lower cell voltage of 1.56 V at 10 mA cm^−2^ was reported for HfNiSe_2_/rGO, indicating highly efficient combined HER and OER activity and excellent stability [135].

Overall, the data summarized in Table 8 demonstrate that noble-metal-based catalysts such as Ru-containing systems provide the lowest HER overpotentials, whereas transition-metal compounds based on Ni, Fe, Mo, and P offer competitive catalytic activity combined with significantly improved durability and economic feasibility. Consequently, heterostructured and doped transition-metal materials are emerging as promising candidates for scalable AEM water electrolysis technologies.

**Figure 14:**
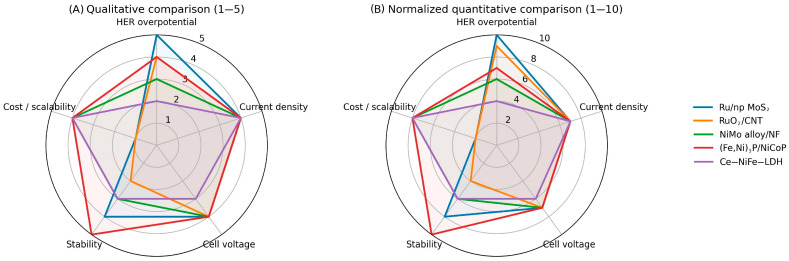
Comparative performance of electrocatalysts for anion exchange membrane (AEM) water electrolysis. (**A**) Qualitative comparison on a scale from 1 (poor) to 5 (excellent). (**B**) Normalized comparison on a scale from 2 (poor) to 10 (excellent). The evaluated parameters include catalytic activity (HER/OER overpotential at 10 mA cm^−2^), achievable current density, energy efficiency, operational stability, and cost/scalability. Scores are derived from normalized values reported in representative studies [98,110,113,129,130,131]. Activity values represent either HER or OER performance depending on the reported reaction for each catalyst. Stability categories correspond to approximate operational ranges (<50 h, 50–200 h, >200 h), while cost/scalability reflects both material composition (e.g., noble metal content) and synthesis complexity. The radar plots provide a semi-quantitative comparison of performance trade-offs and are intended for comparative visualization rather than absolute quantitative assessment.

The radar plots in Figure 14 present a comparative assessment of five representative electrocatalysts used in alkaline membrane electrolysis systems, taking into account both catalytic activity and practical parameters such as durability and scalability.

In panel A, a qualitative evaluation of the catalytic properties is shown. Among the analyzed materials, Ru/np-MoS_2_ exhibits the highest HER activity, achieving a very low overpotential of approximately 30 mV at 10 mA cm^−2^ together with favorable reaction kinetics, which results in the highest score in the HER overpotential category [130]. However, its practical implementation may be limited by the high cost associated with the use of ruthenium. A similar trend is observed for the RuO_2_/CNT paper electrode, which also demonstrates high catalytic activity but receives a lower rating in the cost and scalability category due to the presence of noble metals [129].

Transition-metal-based catalysts such as NiMo alloy/NF and (Fe,Ni)_3_P/NiCoP heterointerface show a more balanced performance profile. In particular, (Fe,Ni)_3_P/NiCoP stands out because of its excellent operational stability exceeding 500 h, which is reflected in the highest stability score among the compared catalysts [131]. NiMo alloy/NF provides a favorable compromise between catalytic activity and material cost, making it attractive for potential large-scale hydrogen production systems [98,110].

In panel B, the normalized quantitative comparison further highlights these trends. Ruthenium-containing catalysts, especially Ru/np-MoS_2_, reach the highest values in terms of HER activity due to their extremely low overpotential [130]. Nevertheless, non-noble-metal catalysts such as Ce-doped NiFe-LDH demonstrate competitive stability and significantly improved cost and scalability potential, despite requiring somewhat higher overpotentials for oxygen evolution [113].

Overall, the radar-plot comparison clearly illustrates a trade-off between catalytic activity and material cost. While noble-metal-based catalysts provide superior electrochemical performance, transition-metal systems based on Ni, Fe, and Mo offer a more balanced combination of activity, stability, and economic feasibility, making them particularly promising candidates for scalable AEM water electrolysis technologies.

Overall, the development of electrode materials for AEM electrolysis is increasingly focused on advanced transition-metal catalysts and engineered nanostructures that combine high catalytic activity for HER and OER with improved durability and cost-effectiveness, while emerging strategies such as single-atom catalysts and heterostructured materials offer promising pathways toward highly efficient and scalable alkaline water electrolysis systems [98,110,113,129,130,131,132,133,134,135].

### 3.5. Emerging Materials

Recent progress in electrocatalytic materials for hydrogen production has expanded beyond conventional catalysts such as graphene and perovskites toward a wide range of advanced materials, including two-dimensional (2D) materials, high-entropy compounds, transition-metal nitrides, chalcogenides, and hybrid heterostructures. These materials are intensively investigated due to their ability to combine high catalytic activity, structural stability, and cost-effective synthesis, which are essential for large-scale hydrogen production technologies [100,136,137].

#### 3.5.1. Two-Dimensional Materials Beyond Graphene

Two-dimensional materials, including transition-metal dichalcogenides (TMDs), MXenes, and emerging metal borides (MBenes), have attracted considerable attention for HER and OER catalysis due to their high surface area and abundance of exposed active sites [123,138]. Representative systems typically achieve current densities of 10–100 mA cm^−2^ at HER overpotentials of ~50–200 mV in alkaline or acidic electrolytes, with Tafel slopes in the range of 40–100 mV dec^−1^, depending on composition and structure.

The electronic properties of 2D catalysts can be tuned through defect engineering, heteroatom doping, and heterostructure formation, enabling optimization of hydrogen adsorption energy and charge-transfer kinetics [136,138]. For example, MXene-based composites and MoS_2_-derived heterostructures show enhanced performance due to improved conductivity and interfacial charge transfer, often maintaining stable operation for 20–100 h under typical conditions [130,133]. Compared to conventional transition-metal catalysts, 2D materials offer improved active-site accessibility and tunability, although their long-term stability and scalability remain key challenges.

#### 3.5.2. High-Entropy and Multi-Component Materials

High-entropy materials (HEMs), including high-entropy alloys (HEAs) and high-entropy oxides (HEOs), represent an emerging class of electrocatalysts characterized by multi-element compositions and complex electronic structures [139,140]. These systems typically exhibit HER and OER overpotentials in the range of 100–300 mV at 10 mA cm^−2^, with Tafel slopes of 30–80 mV dec^−1^, depending on the specific composition and electrolyte.

The high configurational entropy contributes to enhanced structural stability and corrosion resistance, particularly under alkaline conditions, where many transition-metal-based systems demonstrate improved durability compared to acidic environments [141,142]. Stability tests often indicate operation over 50–200 h with limited performance degradation, highlighting their potential for practical applications. Recent studies demonstrate that HEOs and selenides exhibit excellent catalytic activity for both HER and OER due to optimized electronic structures and synergistic interactions among the constituent elements [134,140]. Compared to conventional binary or ternary catalysts, HEMs provide a broader compositional space for tuning catalytic activity through synergistic interactions. However, challenges related to controlled synthesis, compositional optimization, and mechanistic understanding still limit their widespread implementation.

#### 3.5.3. Bifunctional and Hybrid Nanostructures

Bifunctional electrocatalysts capable of driving both HER and OER are essential for efficient overall water splitting. Hybrid nanostructures based on transition-metal nitrides, phosphides, oxides, and chalcogenides typically achieve overall water splitting at cell voltages of ~1.5–1.8 V at 10 mA cm^−2^, with individual HER and OER overpotentials in the range of 50–300 mV depending on the system [124,143].

These materials benefit from synergistic effects at heterointerfaces, which enhance charge transfer and optimize adsorption energies of reaction intermediates. For example, cobalt-based heterostructures and ternary chalcogenides demonstrate improved catalytic performance, with Tafel slopes typically between 40 and 90 mV dec^−1^ and operational stability exceeding 50–150 h under alkaline conditions [112,135].

Compared to single-phase catalysts, hybrid systems offer improved activity and durability due to interfacial effects; however, their performance strongly depends on electrolyte conditions and structural stability, highlighting the importance of rational interface engineering.

#### 3.5.4. Integrated Analysis: Stability, Electrolyte Effects, and Design Trade-Offs

Electrocatalytic performance in water electrolysis is governed by a complex interplay between catalyst composition, electrolyte environment, and operating conditions, making direct comparisons across systems inherently system-dependent [25,32,39]. Key performance metrics, including current density, overpotential, Tafel slope, and stability, must therefore be evaluated within the context of the specific electrolysis technology.

Transition-metal-based catalysts generally exhibit high activity and durability in alkaline media, where they can operate at current densities of 10–100 mA cm^−2^ with overpotentials in the range of 100–300 mV and maintain stability over 50–200 h [42,43,44,45,97,98,99,100,101,102]. However, their performance is significantly compromised in acidic environments due to corrosion and dissolution, limiting their applicability in PEM systems [49,50,51,52,53,55]. In contrast, noble-metal-based catalysts, such as Pt, IrO_2_, and RuO_2_, demonstrate superior stability and lower overpotentials (<100 mV for HER) under acidic conditions, albeit at the expense of higher cost and limited availability [25,52,53,54,55,56].

The electrolyte plays a decisive role in determining both catalytic activity and long-term durability. Alkaline electrolytes (e.g., KOH) enable the use of earth-abundant materials but typically require higher overpotentials for OER (200–400 mV) due to slower reaction kinetics [42,43,44,45,47]. Acidic environments, as in PEM electrolysis, provide faster proton transport and improved HER kinetics but impose strict material constraints, restricting catalyst selection to corrosion-resistant noble metals [49,50,51,52,53,55,56]. These observations highlight that catalyst performance cannot be decoupled from the reaction environment and must be evaluated in a system-specific framework [25,51].

Electrode–electrolyte interface instability is a key degradation mechanism in electrolysis systems. It involves processes such as catalyst dissolution, corrosion, phase transformation, and interfacial delamination [25,52,53]. In PEM systems, instability is often associated with noble metal dissolution and membrane degradation under high anodic potentials [52,53,54,55,56]. In ALK systems, surface reconstruction and oxide formation may alter catalytic activity over time [42,45]. In SOEC systems, thermomechanical stress and interdiffusion at interfaces contribute to long-term degradation [61,62,63,64,65]. Understanding these mechanisms is essential for improving catalyst durability and ensuring stable long-term operation under industrial conditions.

A central challenge in electrocatalyst development is the trade-off between activity, stability, and cost [11,12,33]. While noble metals offer the highest intrinsic activity and stability, their economic limitations hinder large-scale deployment. Transition-metal-based catalysts provide a cost-effective alternative but often require higher overpotentials and exhibit reduced stability under certain conditions, particularly in acidic media [42,43,44,45,49,50,51]. Hybrid and multi-component systems, including heterostructures and high-entropy materials, partially address these limitations by leveraging synergistic effects to enhance both activity and durability [134,140,143].

From a design perspective, future progress requires a shift from isolated performance optimization toward integrated, application-oriented strategies. Approaches such as defect engineering, interface design, and compositional tuning have demonstrated significant improvements in catalytic performance; however, their effectiveness is strongly dependent on operating conditions, including electrolyte composition, temperature, and current density [136,138,140,143]. Importantly, industrially relevant operation requires current densities exceeding 200 mA cm^−2^ and long-term stability beyond 100 h, emphasizing the need for scalable and robust materials [33,39,86,87,88,89,90].

These findings demonstrate that no single class of electrocatalysts is universally optimal; instead, their rational design must be guided by a system-specific balance between activity, stability, and cost, supported by standardized evaluation protocols and cross-system comparisons to enable scalable and durable water electrolysis technologies [25,34].

Despite significant progress in catalyst development, scaling up electrocatalysts for industrial water electrolysis remains a major challenge. Many high-performance materials are synthesized using complex laboratory-scale methods that are difficult to translate into large-scale production [33,39]. Key limitations include maintaining uniform catalyst structure, ensuring reproducibility, reducing synthesis cost, and achieving long-term stability under industrial operating conditions. Furthermore, integration of advanced catalysts into practical electrode architectures and membrane–electrode assemblies represents a critical bottleneck for commercialization.

Although substantial progress has been achieved between 2021 and 2025, several limitations remain in the current research landscape. Many studies focus on laboratory-scale performance under idealized conditions, which do not fully reflect industrial operation [29,34]. In addition, the lack of standardized testing protocols and inconsistent reporting metrics complicates direct comparison between studies. Limited long-term stability data and insufficient attention to scalability further restrict the practical applicability of reported electrocatalysts. Addressing these limitations is essential for bridging the gap between fundamental research and industrial deployment.

In this context, machine learning and data-driven approaches are emerging as powerful tools for accelerating catalyst discovery and optimization. By analyzing large datasets of experimental and computational results, machine learning models can identify structure–property relationships and predict catalytic performance [35,36,37]. These approaches enable rapid screening of candidate materials and facilitate the design of catalysts with optimized activity, stability, and cost. Integration of machine learning with experimental and theoretical methods is expected to significantly accelerate the development of next-generation electrode materials for green hydrogen production.

## 4. Conclusions

This review systematically analyzed recent progress in electrode materials across four major water electrolysis technologies: ALK, PEM, SOEC, and AEM systems. Despite sharing the same fundamental reaction—the electrochemical splitting of water—each technology operates under distinct physicochemical conditions that impose different requirements on electrode composition, catalytic activity, stability, and structural design.

Several key conclusions can be drawn from the comparative analysis:

First, catalyst design strategies are increasingly shifting toward multicomponent and heterostructured materials. Interfaces, defect engineering, strain effects, and electronic structure modulation significantly influence adsorption energies of reaction intermediates, enabling improved catalytic activity for both the HER and OER.

Second, a clear activity–stability–cost trade-off remains a central challenge. Noble-metal catalysts still deliver the highest intrinsic activity, particularly in PEM systems operating in acidic environments. However, their limited availability and high cost drive intensive research into transition-metal-based alternatives and nanostructured architectures that can approach the performance of platinum-group metals while offering superior scalability.

Third, advances in nanostructured catalysts, heterointerfaces, and defect-engineered materials have enabled transition-metal systems to achieve remarkable catalytic performance, particularly in alkaline environments. Such materials represent promising candidates for industrial-scale electrolysis due to their lower cost and improved material abundance.

Fourth, durability and long-term operational stability remain critical barriers to commercialization. Degradation processes—including catalyst dissolution, structural coarsening, membrane degradation, and electrode–electrolyte interface instability—are strongly dependent on operating conditions and system architecture. Consequently, understanding degradation mechanisms through operando characterization and multiscale modeling is essential for future electrolyzer development.

From a technological perspective, the future hydrogen economy will likely rely on a diversified portfolio of electrolysis technologies rather than a single dominant system. Mature ALK electrolysis will remain important for large-scale hydrogen production due to its robustness and low capital costs. PEM electrolysis offers superior dynamic response and compatibility with intermittent renewable energy sources. High-temperature SOEC systems provide outstanding thermodynamic efficiency when integrated with industrial heat sources, while emerging AEM electrolysis represents a promising pathway toward cost-effective, PGM-free systems.

Looking forward, several research directions appear particularly important: (i) development of PGM-free catalysts with industrial-level current densities; (ii) improved membrane–electrode interface engineering; (iii) scalable synthesis routes for advanced electrocatalysts; (iv) integration of computational materials design and machine learning, and (v) standardized testing protocols under industrial operating conditions. Such protocols are essential for the reliable comparison of electrocatalyst performance. Key parameters include current density benchmarks (e.g., 10, 100, 500 mA cm^−2^), long-term stability tests under constant current or potential, and operation under realistic conditions such as elevated temperature and pressure [33,34]. The lack of standardized evaluation methods in the literature complicates direct comparison between studies and limits the transferability of laboratory-scale results to industrial systems. Therefore, future research should focus on developing unified testing frameworks aligned with practical operating conditions.

Ultimately, progress in electrode materials will play a decisive role in reducing hydrogen production costs and enabling the large-scale deployment of green hydrogen technologies. Continued interdisciplinary collaboration between materials science, electrochemistry, and system engineering will therefore be essential to translate laboratory discoveries into durable, efficient, and economically viable electrolysis systems.

This review highlights the critical role of balancing catalytic activity, long-term stability, and material cost in the development of next-generation electrode materials for scalable hydrogen production [33,39,86,87,88,89,90].

Future research on electrode materials will increasingly focus on integrating catalyst design with system-level requirements. Key priorities include improving long-term durability, reducing reliance on critical raw materials, and developing scalable synthesis strategies. In addition, designing catalysts capable of operating efficiently under dynamic conditions associated with renewable energy sources will be essential. The development of multifunctional materials with adaptive surface properties represents a promising direction for next-generation electrolysis systems [33,39,86,87,88,89,90,112,144,145,146,147,148,149].

## Figures and Tables

**Table 1:** Key material requirements for electrodes in ALK water electrolysis.

Property	Technical Requirement	Representative Materials	Refs.
Corrosion Resistance	Chemical stability in 25–40 wt% KOH/NaOH; resistance to passivation and surface oxidation	Ni, NiFe, NiCo oxides, stainless steel	[25,42,43,45]
Electrical Conductivity	High bulk and interfacial conductivity; low ohmic losses	Ni foam, Ni mesh, Ni plates	[43,45,46]
Catalytic Activity (HER)	Low HER overpotential; optimized hydrogen adsorption energy	NiMo alloys, Ni-based composites	[43,44,45]
Catalytic Activity (OER)	Low OER overpotential; enhanced oxygen intermediate adsorption/desorption	NiFeOx, NiFe LDH	[42,45,47,48]
Electrochemical Stability	Long-term durability at >200 mA cm^−2^; resistance to dissolution and structural degradation	Ni-based alloys, reinforced composites	[43,45,46]
Mechanical Integrity	Structural robustness under gas evolution and pressure fluctuations	Porous metallic substrates	[13,45,46]
Cost Effectiveness	Use of earth-abundant, non-noble metals	Transition metal alloys	[2,42,43,45]
Material Availability	Scalable raw material supply	Ni-based systems	[2,30,45]

Notes: HER—hydrogen evolution reaction; OER—oxygen evolution reaction; LDH—layered double hydroxide.

**Table 2:** Key material requirements for electrodes in PEM water electrolysis.

Property	Technical Requirement	Representative Materials	Refs.
Proton Conductivity (Interface)	High proton conductivity and low interfacial resistance at catalyst–membrane interface	PFSA (e.g., Nafion)-based catalyst layers; ionomer-modified CLs	[49,50,52,53]
Acidic Stability	Chemical and electrochemical stability at pH < 1 under high anodic potentials	IrO_2_, RuO_2_, mixed Ir-based oxides	[52,53,54]
Catalytic Activity	Ultra-low overpotential and high exchange current density for HER/OER	Pt (HER); IrO_2_ (OER); Pt-based nanostructures	[25,53,54,55,56]
Corrosion Resistance	Resistance to oxidative dissolution and passivation at >1.6 V vs. RHE	Ti substrates, Ti felt, noble metal coatings	[45,52,53]
Thermal Stability	Stable electrochemical performance at 50–80 °C under dynamic load	Noble metal catalysts; thermally stable PFSA membranes	[45,49,53]
Mechanical Integrity	Resistance to compression, swelling, and differential pressure (up to 30–40 bar)	Porous transport layers (PTL); Ti felt; reinforced PFSA membranes	[49,51,52]
Membrane Compatibility	Chemical compatibility with PFSA membranes and minimal ionomer degradation	PFSA-compatible catalysts; optimized ionomer distribution	[49,50,52]
Cost & Critical Raw Materials	Reduced noble metal loading while maintaining activity and durability	Low-Ir catalysts; sputtered thin films; core–shell architectures	[11,12,33,41,52,56]

Notes: PFSA—perfluorosulfonic acid; CLs—catalyst layers; HER—hydrogen evolution reaction; OER—oxygen evolution reaction; RHE—reversible hydrogen electrode; PTL—porous transport layer.

**Table 3:** Key material requirements for electrodes in SOEC water electrolysis.

Property	Technical Requirement	Representative Materials	Refs.
Oxygen-Ion Conductivity	Fast O^2−^ transport through electrolyte and across electrode–electrolyte interface	YSZ, GDC (Gd-doped ceria)	[59,60,65]
Thermal Expansion Compatibility	Matched thermal expansion coefficients (TEC) between electrolyte and electrodes	Ni–YSZ cermets, LSM	[60,62,71]
Redox Stability	Stability under alternating reducing/oxidizing atmospheres and transient operation	Perovskites (LSM, LSCF), doped ceria systems	[61,63,68]
Catalytic Activity	High catalytic activity for H_2_O and CO_2_ reduction (fuel electrode) and O_2_ evolution (oxygen electrode)	Ni–YSZ (fuel), LSCF (oxygen)	[59,65,67]
Thermal Shock Resistance	Resistance to rapid heating/cooling cycles and thermal gradients	Ceramic composites, graded structures	[62,68]
Chemical Compatibility	No detrimental interfacial reactions or secondary phase formation	Doped zirconia systems, barrier layers (GDC)	[60,64]
Electrical Conductivity	High electronic or mixed ionic–electronic conductivity at 600–850 °C	Mixed ionic–electronic conductors (MIEC), perovskites	[59,67,71]
Mechanical Stability	Structural integrity during long-term high-temperature operation and pressurization	Ceramic-supported cells, robust porous backbones	[62,69]
Cost & Scalability	Use of non-noble, abundant ceramic materials; scalable fabrication methods	Ni-based cermets, perovskite oxides	[32,67,72]

Notes: YSZ—yttria-stabilized zirconia; GDC—gadolinium-doped ceria; TEC—thermal expansion coefficient; LSM—lanthanum strontium manganite; LSCF—lanthanum strontium cobalt ferrite; MIEC—mixed ionic–electronic conductor.

**Table 4:** Key material requirements for electrodes in AEM water electrolysis.

Property	Technical Requirement	Representative Materials	Refs.
Hydroxide-Ion Conductivity	Efficient OH^−^ transport across membrane–electrode interface	Quaternary ammonium-based AEM polymers	[74,77,81]
Alkaline Stability	Resistance to nucleophilic attack by OH^−^	Crosslinked and chemically stabilized AEMs	[75,83,84]
Catalytic Activity	Low overpotential under alkaline conditions	NiFeOx, Co-based catalysts, perovskites	[42,43,44,78]
Gas Barrier Properties	Low H_2_/O_2_ crossover	Dense polymer AEM membranes	[75,83]
Mechanical Robustness	Stability under pressure and hydration cycles	Reinforced AEM membranes	[77,81]
Thermal Stability	Stable operation at 50–80 °C	Stabilized polymer backbones	[74,80]
Membrane Compatibility	Chemical compatibility with ionomer binder	AEM-compatible catalyst layers	[81,85]
Cost & Abundance	Elimination of noble metals (PGMs)	Non-PGM catalysts (Ni-based, Raney Ni)	[74,79]

Notes: AEM—anion exchange membrane; PGMs—platinum group metals.

**Table 5:** Selected high-performance HER and OER electrocatalysts for ALK electrolysis.

Electrocatalyst	Reaction	Electrolyte	j [mA cm^−2^] @ η [mV] or Cell Voltage [V]	Tafel Slope [mV dec^−1^]	Stability	Refs.
PdPtRhRuAu HEAA	HER	1 M KOH	10 @ 12	17	160 h	[100]
Pt_34_Co_66_ thin film	HER	1 M KOH + NaCl	10 @ 30	–	24 h	[101]
MoNi_4_/MoO_2_@Ni	HER	1 M KOH	500 @ 65	30	–	[102]
Pt/Ni–Mo	HER	alkaline (KOH-based)	2000 @ 113	–	140 h at 2000 mA cm^−2^, up to 80 °C	[102]
FeIr/NF	HER	alkaline (KOH-based)	500–3500 @ 125–471	–	504 h at 150 mA cm^−2^	[102]
Ru–O–Mo (Ru/MoO_2_)	HER	1 M KOH	10 @ 16	32	40 h	[103]
Ni(OH)_2_/Ni_3_N/NF	HER	KOH (Fe impurity)	50–100 @ 172–207	100	after 50 activation cycles	[104]
CoFe_2_O_4_@Ni_2_P/NF	HER	1 M KOH	10 @ 55	–	good cycling stability	[105]
CoFe_2_O_4_@Ni_2_P/NF	OER	1 M KOH	100 @ 261	–	good cycling stability	[105]
CoFe_2_O_4_@Ni_2_P/NF	HER/OER (overall water splitting)	1 M KOH	10 @ 1.60 V	–	good stability	[105]
NiO@MoO_3−*x*_/Ni	HER	1 M KOH	10 @ 7 100 @ 75 200 @ 112	34	40 h at 100 mA cm^−2^	[106]
NiO@MoO_3−*x*_/Ni	HER/OER (overall water splitting)	1 M KOH	10 @ 1.47 V	–	–	[106]
Fe-hydroxide@CoS	OER	1 M KOH	10 @ 270	46	high durability	[107]
Ir_2_P/Ru_2_P hollow nanotubes	HER	1 M KOH	10 @ 23	31	50 h	[108]
V@CoSe_2_	HER	1 M KOH	10 @ 212	94	40 h	[109]
V@CoSe_2_	OER	1 M KOH	10 @ 310	52	16 h	[109]
V@CoSe_2_	HER/OER (overall water splitting)	1 M KOH	10 @ 1.96 V (cell voltage)	–	>24 h	[109]
Ni_3_S_2_/NF	HER	1 M KOH	20 @ 48	–	40 h	[110]
Pt cluster- NiCoP@NF NWs	HER	1 M KOH	100 @ 65	39	5000 cycles; 30 h @ 500 mA cm^−2^	[111]
Co_3_Mo_3_N/Co_4_N/Co	HER	1 M KOH	10 @ 58	39	long-term stable	[112]
Co_3_Mo_3_N/Co_4_N/Co	OER	1 M KOH	10 @ 270	52	long-term stable	[112]
Co_3_Mo_3_N/Co_4_N/Co	HER/OER (overall water splitting)	1 M KOH	10 @ 1.58 V (cell voltage)	–	100 h @ 200 mA cm^−2^ (~100% retention)	[112]
NiFe@NiCr-LDH	OER	1 M KOH	10 @ 266	63	10% current decay after 20 h	[113]

Notes: HER—hydrogen evolution reaction; OER—oxygen evolution reaction; η—overpotential; j—current density; HEAAs –high-entropy alloy aerogels; NF—nickel foam; NWs—nanowires; LDH—layered double hydroxide.

**Table 6:** Selected high-performance HER and OER electrocatalysts for PEM electrolysis.

Electrocatalyst	Reaction	Electrolyte	j [mA cm^−2^] @ η [mV] or Cell Voltage [V]	Tafel Slope [mV dec^−1^]	Stability	Refs.
TS–Ir/MnO_2_	OER	0.1 M HClO_4_	10 @ 198	56.6	200 h @ 500 mA cm^−2^	[114]
RuIr-NC (Ru–Ir nanosheets)	HER	0.5 M H_2_SO_4_	10 @ 46	32	>120 h @ 10 mA cm^−2^	[116]
RuIr-NC	OER	0.5 M H_2_SO_4_	10 @ 165	40	>122 h @ 1 mA cm^−2^	[116]
RuIr-NC‖RuIr-NC	HER/OER (overall water splitting)	0.5 M H_2_SO_4_	10 @ 1.485 V (cell voltage)	—	>120 h	[116]
Sr_2_CaIrO_6_ (Ir double perovskite)	OER	0.1 M HClO_4_	10 @ 250	35	450 h @ 2 A cm^−2^ (PEMWE conditions)	[117]
Ru_6_W_4_O_x_-400 °C	OER	0.5 M H_2_SO_4_	10 @ 140	51.4	150 h	[118]
IrO_2_/TiO_2_ (Benchmark, 2.0 mg_Ir_ cm^−2^)	OER	PEM (Nafion^®^ 117, acidic, ultrapure H_2_O feed, 60 °C; Ir loading: 2.0 mg_Ir_ cm^−2^ (IrO_2_/TiO_2_) or 0.25 mg_Ir_ cm^−2^ (a-IrO(OH)x/TiO_2_))	1500 @ 1.79 V	52 → 72	3700 h (stable after initial degradation)	[119]
a-IrO(OH)_x_/TiO_2_ (P2X, 0.25 mg_Ir_ cm^−2^)	OER	PEM (Nafion^®^ 117, acidic, ultrapure H_2_O feed, 60 °C; Ir loading: 2.0 mg_Ir_ cm^−2^ (IrO_2_/TiO_2_) or 0.25 mg_Ir_ cm^−2^ (a-IrO(OH)_x_/TiO_2_))	1500 @ 1.79 V	45 → 61	3700 h (higher initial decay, then stable)	[119]
Pt/C (0.3 mg_Pt_ cm^−2^)	HER	PEM (Nafion^®^ 117, acidic, ultrapure H_2_O feed, 60 °C; Ir loading: 2.0 mg_Ir_ cm^−2^ (IrO_2_/TiO_2_) or 0.25 mg_Ir_ cm^−2^ (a-IrO(OH)x/TiO_2_))	1000 @ 5	30	stable (not limiting)	[119]
IrO_x_/Ti_4_O_7_ (P2X) Reduced Ir	OER	PEM (Nafion^®^, acidic, ultrapure H_2_O feed, ~80 °C; Ir loading: ~0.3–0.5 mg_Ir_ cm^−2^)	~1000 @ ~1.75–1.80 V	50–60	stable >50 h @ 1 A cm^−2^ improved Ir utilization vs. TiO_2_	[120]
Core–shell IrCo@IrCoO_x_	OER	0.5 M H_2_SO_4_	10 @ 259	59	55 h @ 50 mA cm^−2^	[121]
Ru_1_/NiCo LDH Atomically dispersed Ru	HER/OER (bifunctional)	1.0 M KOH	HER: 10 @ 18; OER: 10 @ 189; overall: 10 @ 1.45 V	29	stable (≥100 h, negligible decay; 2000 cycles)	[122]
MBenes/MXene-derived (transition metal borides, e.g., Mo_2_B_2_, Ti_2_B_2_, doped systems)	HER/OER (overall water splitting)	0.5 M H_2_SO_4_/1.0 M KOH (depending on system)	10 @ 1.45–1.60 V	40–120	10–100 h	[123]
TM sulfides/phosphides (general class)	HER & OER	0.5 M H_2_SO_4_/1.0 M KOH	10 @ 20–150	30–150	10–200 h (depending on material)	[124]

Notes: PEMWE—proton exchange membrane water electrolysis; HER—hydrogen evolution reaction; OER—oxygen evolution reaction; η—overpotential; j—current density; NC—nanosized coral; TS—tensile-strained; LDH—layered double hydroxide; TM—transition metal; MXene—two-dimensional transition metal carbides/nitrides; MBenes—two-dimensional transition metal borides.

**Table 7:** Selected high-performance HER and OER electrocatalysts for SOEC electrolysis.

Electrocatalyst	Reaction	Electrolyte	j [mA cm^−2^] @ η [V] or Cell Voltage [V]	Tafel Slope [mV dec^−1^]	Stability	Refs.
Ni–YSZ cermet	HER (steam reduction)	YSZ	1000 @ 1.30 (800 °C)	120	>1000 h	[65,69]
Ni–YSZ (co-electrolysis conditions)	HER	YSZ	1000 @ 1.35 (800 °C)	115	1000 h	[62,65]
Ni–GDC composite	HER	GDC	700 @ 1.25 (750 °C)	105	500 h	[66,69]
LSM (La_0.8_Sr_0.2_MnO_3_)	OER	YSZ	500 @ 1.35 (800 °C)	90	>1000 h	[59,69]
LSCF (La_0.6_Sr_0.4_Co_0.2_Fe_0.8_O_3_)	OER	GDC	1000 @ 1.30 (750 °C)	75	800 h	[61,69]
LSC (La_0.6_Sr_0.4_CoO_3_)	OER	ScSZ	800 @ 1.28 (750 °C)	70	600 h	[66,69]
LSM–YSZ composite	OER	YSZ	600 @ 1.32 (800 °C)	85	>900 h	[69]
Ni–ScSZ cermet	HER	ScSZ	900 @ 1.28 (800 °C)	110	700 h	[60,69]
Pr_2_Ni_0.8_Co_0.2_O_4_+δ (Ruddlesden–Popper)	OER	YSZ/GDC	900 @ 1.30 (700–750 °C)	65	>700 h	[69]
La_2_NiO_4_+δ nanostructured electrode	OER	YSZ	600 @ 1.25 (700 °C)	60	500 h	[69]
LSCF–GDC composite electrode	OER	YSZ + GDC barrier	880 @ 1.30 (750 °C)	70	>500 h	[125]
Ru-infiltrated LSCF	OER	YSZ	656 @ 1.30 (700 °C)	65	>400 h	[126]
LSFN (La_0.9_Sr_0.1_Fe_0.9_Nb_0.1_O_3_-δ)	HER	GDC/YSZ	1516 @ 1.5 (850 °C)	80	60 h	[127]
BLF (Ba_0.95_LaFeO_3_-δ)	OER	YSZ	3170 @ 1.5 (850 °C)	75	200 h	[128]
LSCM–GDC composite (La_0.75_Sr_0.25_Cr_0.5_Mn_0.5_O_3_)	HER	GDC	2360 @ 1.5 (800 °C)	85	200 h	[128]

Notes: HER—hydrogen evolution reaction; OER—oxygen evolution reaction; j—current density; YSZ—yttria-stabilized zirconia; GDC—gadolinium-doped ceria; ScSZ—scandia-stabilized zirconia; Ni–YSZ—nickel–yttria-stabilized zirconia cermet; Ni–GDC—nickel–gadolinium-doped ceria composite; Ni–ScSZ—nickel–scandia-stabilized zirconia cermet; LSM—lanthanum strontium manganite; LSCF—lanthanum strontium cobalt ferrite; LSC—lanthanum strontium cobaltite; LSM–YSZ—lanthanum strontium manganite–yttria-stabilized zirconia composite; LSCF–GDC—lanthanum strontium cobalt ferrite–gadolinium-doped ceria composite; LSFN—lanthanum strontium iron niobate; BLF—barium lanthanum ferrite; LSCM—lanthanum strontium chromium manganite; δ—oxygen non-stoichiometry parameter in oxide lattices; Ruddlesden–Popper—layered perovskite oxide structure (A_2_BO_4_-type).

**Table 8:** Selected high-performance HER and OER electrocatalysts for AME electrolysis.

Electrocatalyst	Reaction	Electrolyte	j [mA cm^−2^] @ η [mV] or Cell Voltage [V]	Tafel Slope [mV dec^−1^]	Stability	Refs.
NiMo alloy/NF	HER	1 M KOH	10 @ 60	55	>24 h @ 10 mA cm^−2^	[98,110]
Ru/np MoS_2_ (single-atom Ru on nanoporous MoS_2_)	HER	1 M KOH	10 @ 30	31	>100 h @ 10 mA cm^−2^	[130]
(Fe,Ni)_3_P/NiCoP heterointerface	HER	1 M KOH	10 @ 52.3	84	>500 h	[131]
Ce-doped NiFe-LDH	OER	1 M KOH	10 @ 240–260	59	50 h @ 10 mA cm^−2^	[113]
RuO_2_/CNT paper electrode	HER (AEMWE cathode)	1 M KOH	1000 @ 1.8 V	30–40	>100 h	[129]
Ni NPs/GDY (graphdiyne-supported nickel nanoparticles)	OER	1 M KOH	10 @ 294	56.8	90 h @ 10 mA cm^−2^	[132]
NHCoMX (Ti_3_C_2_ MXene/Co–Ni(OH)_2_)	HER	1 M KOH	10 @ 73	75	50 h @ 10 mA cm^−2^	[133]
NHCoMX (Ti_3_C_2_ MXene/Co–Ni(OH)_2_)	OER	1 M KOH	10 @ 270	65	50 h @ 10 mA cm^−2^	[133]
NHCoMX (Ti_3_C_2_ MXene/Co–Ni(OH)_2_)	HER/OER (overall water splitting)	1 M KOH	10 @ 1.55–1.60 V (cell voltage)	—	50 h @ 10 mA cm^−2^	[133]
NiFeSe/CFP (NiFe selenide on carbon fiber paper)	HER	1 M KOH	10 @ 186	52	20 h @ 10 mA cm^−2^	[134]
HfNiSe_2_/rGO (hafnium–nickel selenide on rGO)	HER/OER (overall water splitting)	1 M KOH	10 @ 1.56 V (cell voltage)	—	>100 h @ 10 mA cm^−2^	[135]

Notes: HER—hydrogen evolution reaction; OER—oxygen evolution reaction; η—overpotential; j—current density; AEMWE—anion exchange membrane water electrolysis; NF—nickel foam; np—nanoporous; LDH—layered double hydroxide; CNT—carbon nanotubes; GDY—graphdiyne; MXene—Ti_3_C_2_ MXene; NPs—nanoparticles; CFP—carbon fiber paper; rGO—reduced graphene oxide.

## Data Availability

No new data were created or analyzed in this study. Data sharing is not applicable to this article.

## Abbreviations

The following abbreviations are used in this manuscript:

AEM	Anion Exchange Membrane
AEMWE	Anion Exchange Membrane Water Electrolysis
ALK	Alkaline Water Electrolysis
BLF	Barium Lanthanum Ferrite
BoP	Balance of Plant
CAGR	Compound Annual Growth Rate
CCU	Carbon Capture and Utilization
CLs	Catalyst Layers
CNT	Carbon Nanotube
DC	Direct Current
DFT	Density Functional Theory
DSA	Dimensionally Stable Anode
EIS	Electrochemical Impedance Spectroscopy
GDC	Gadolinium-Doped Ceria
HER	Hydrogen Evolution Reaction
HEM	High-Entropy Material
HEA	High-Entropy Alloy
HEAA	High-Entropy Alloy Aerogel
HEO	High-Entropy Oxide
LCA	Life Cycle Assessment
LDH	Layered Double Hydroxide
LOM	Lattice Oxygen–Mediated Mechanism
LSC	Lanthanum Strontium Cobaltite
LSCM	Lanthanum Strontium Chromium Manganite
LSCF	Lanthanum Strontium Cobalt Ferrite
LSF	Lanthanum Strontium Ferrite
LSFN	Lanthanum Strontium Iron Niobate
LSM	Lanthanum Strontium Manganite
MBene	Two-Dimensional Transition Metal Boride
MEA	Membrane Electrode Assembly
MIEC	Mixed Ionic–Electronic Conductor
MXene	Two-Dimensional Transition Metal Carbide/Nitride
NC	Nanosized Coral
NF	Nickel Foam
NWs	Nanowires
OER	Oxygen Evolution Reaction
PEM	Proton Exchange Membrane
PEMWE	Proton Exchange Membrane Water Electrolysis
PFSA	Perfluorosulfonic Acid
PGM	Platinum Group Metal
PGM-free	Platinum Group Metal-Free
PtX	Power-to-X (conversion of electricity into fuels or chemicals)
PTL	Porous Transport Layer
RES	Renewable Energy Sources
RHE	Reversible Hydrogen Electrode
ScSZ	Scandia-Stabilized Zirconia
SOE	Solid Oxide Electrolysis
SOEC	Solid Oxide Electrolysis Cell
TEC	Thermal Expansion Coefficient
TM	Transition Metal
TMD	Transition Metal Dichalcogenide
TPB	Triple-Phase Boundary
TRL	Technology Readiness Level
TS	Tensile-Strained
YSZ	Yttria-Stabilized Zirconia
